# Coronavirus S protein alters dsRNA accumulation and stress granule formation through regulation of ADAR1-p150 expression

**DOI:** 10.1093/nar/gkae921

**Published:** 2024-10-24

**Authors:** Baochao Fan, Yupeng Li, Yi Wang, Shanshan Yang, Qi Peng, Jiali Qian, Chuanhong Wang, Xue Zhang, Hong Xu, Shiyu Liu, Wenlong He, Gege Zhang, Xuejiao Zhu, Yunchuan Li, Yongxiang Zhao, Mi Hu, Wei Wang, Jinzhu Zhou, Rongli Guo, Kongwang He, Bin Li

**Affiliations:** Institute of Veterinary Medicine, Jiangsu Academy of Agricultural Sciences, Key Laboratory of Veterinary Biological Engineering and Technology, Ministry of Agriculture; Jiangsu Key Laboratory for Food Quality and Safety-State Key Laboratory Cultivation Base of Ministry of Science and Technology, 50 Zhongling Street, Nanjing 210014, China; Jiangsu Co-Innovation Center for Prevention and Control of Important Animal Infectious Diseases and Zoonoses, 88 South Daxue Road, Yangzhou University, Yangzhou 225009, China; College of Veterinary Medicine, Nanjing Agricultural University, 1 Weigang, Xiaolingwei Street, Nanjing 210095, China; School of Life Sciences, Jiangsu University, 301 Xuefu Road, Xiangshan Street, Zhenjiang 212013, China; GuoTai (Taizhou) Center of Technology Innovation for Veterinary Biologicals, 28 Xinglin Road, Taizhou 225300, China; Institute of Veterinary Medicine, Jiangsu Academy of Agricultural Sciences, Key Laboratory of Veterinary Biological Engineering and Technology, Ministry of Agriculture; Jiangsu Key Laboratory for Food Quality and Safety-State Key Laboratory Cultivation Base of Ministry of Science and Technology, 50 Zhongling Street, Nanjing 210014, China; Institute of Veterinary Medicine, Jiangsu Academy of Agricultural Sciences, Key Laboratory of Veterinary Biological Engineering and Technology, Ministry of Agriculture; Jiangsu Key Laboratory for Food Quality and Safety-State Key Laboratory Cultivation Base of Ministry of Science and Technology, 50 Zhongling Street, Nanjing 210014, China; Institute of Veterinary Medicine, Jiangsu Academy of Agricultural Sciences, Key Laboratory of Veterinary Biological Engineering and Technology, Ministry of Agriculture; Jiangsu Key Laboratory for Food Quality and Safety-State Key Laboratory Cultivation Base of Ministry of Science and Technology, 50 Zhongling Street, Nanjing 210014, China; Jiangsu Co-Innovation Center for Prevention and Control of Important Animal Infectious Diseases and Zoonoses, 88 South Daxue Road, Yangzhou University, Yangzhou 225009, China; Institute of Veterinary Medicine, Jiangsu Academy of Agricultural Sciences, Key Laboratory of Veterinary Biological Engineering and Technology, Ministry of Agriculture; Jiangsu Key Laboratory for Food Quality and Safety-State Key Laboratory Cultivation Base of Ministry of Science and Technology, 50 Zhongling Street, Nanjing 210014, China; Jiangsu Co-Innovation Center for Prevention and Control of Important Animal Infectious Diseases and Zoonoses, 88 South Daxue Road, Yangzhou University, Yangzhou 225009, China; Institute of Veterinary Medicine, Jiangsu Academy of Agricultural Sciences, Key Laboratory of Veterinary Biological Engineering and Technology, Ministry of Agriculture; Jiangsu Key Laboratory for Food Quality and Safety-State Key Laboratory Cultivation Base of Ministry of Science and Technology, 50 Zhongling Street, Nanjing 210014, China; College of Veterinary Medicine, Nanjing Agricultural University, 1 Weigang, Xiaolingwei Street, Nanjing 210095, China; Institute of Veterinary Medicine, Jiangsu Academy of Agricultural Sciences, Key Laboratory of Veterinary Biological Engineering and Technology, Ministry of Agriculture; Jiangsu Key Laboratory for Food Quality and Safety-State Key Laboratory Cultivation Base of Ministry of Science and Technology, 50 Zhongling Street, Nanjing 210014, China; Institute of Veterinary Medicine, Jiangsu Academy of Agricultural Sciences, Key Laboratory of Veterinary Biological Engineering and Technology, Ministry of Agriculture; Jiangsu Key Laboratory for Food Quality and Safety-State Key Laboratory Cultivation Base of Ministry of Science and Technology, 50 Zhongling Street, Nanjing 210014, China; Institute of Veterinary Medicine, Jiangsu Academy of Agricultural Sciences, Key Laboratory of Veterinary Biological Engineering and Technology, Ministry of Agriculture; Jiangsu Key Laboratory for Food Quality and Safety-State Key Laboratory Cultivation Base of Ministry of Science and Technology, 50 Zhongling Street, Nanjing 210014, China; Institute of Veterinary Medicine, Jiangsu Academy of Agricultural Sciences, Key Laboratory of Veterinary Biological Engineering and Technology, Ministry of Agriculture; Jiangsu Key Laboratory for Food Quality and Safety-State Key Laboratory Cultivation Base of Ministry of Science and Technology, 50 Zhongling Street, Nanjing 210014, China; College of Veterinary Medicine, Nanjing Agricultural University, 1 Weigang, Xiaolingwei Street, Nanjing 210095, China; Institute of Veterinary Medicine, Jiangsu Academy of Agricultural Sciences, Key Laboratory of Veterinary Biological Engineering and Technology, Ministry of Agriculture; Jiangsu Key Laboratory for Food Quality and Safety-State Key Laboratory Cultivation Base of Ministry of Science and Technology, 50 Zhongling Street, Nanjing 210014, China; Institute of Veterinary Medicine, Jiangsu Academy of Agricultural Sciences, Key Laboratory of Veterinary Biological Engineering and Technology, Ministry of Agriculture; Jiangsu Key Laboratory for Food Quality and Safety-State Key Laboratory Cultivation Base of Ministry of Science and Technology, 50 Zhongling Street, Nanjing 210014, China; Institute of Veterinary Medicine, Jiangsu Academy of Agricultural Sciences, Key Laboratory of Veterinary Biological Engineering and Technology, Ministry of Agriculture; Jiangsu Key Laboratory for Food Quality and Safety-State Key Laboratory Cultivation Base of Ministry of Science and Technology, 50 Zhongling Street, Nanjing 210014, China; Jiangsu Co-Innovation Center for Prevention and Control of Important Animal Infectious Diseases and Zoonoses, 88 South Daxue Road, Yangzhou University, Yangzhou 225009, China; Institute of Veterinary Medicine, Jiangsu Academy of Agricultural Sciences, Key Laboratory of Veterinary Biological Engineering and Technology, Ministry of Agriculture; Jiangsu Key Laboratory for Food Quality and Safety-State Key Laboratory Cultivation Base of Ministry of Science and Technology, 50 Zhongling Street, Nanjing 210014, China; Jiangsu Co-Innovation Center for Prevention and Control of Important Animal Infectious Diseases and Zoonoses, 88 South Daxue Road, Yangzhou University, Yangzhou 225009, China; Institute of Veterinary Medicine, Jiangsu Academy of Agricultural Sciences, Key Laboratory of Veterinary Biological Engineering and Technology, Ministry of Agriculture; Jiangsu Key Laboratory for Food Quality and Safety-State Key Laboratory Cultivation Base of Ministry of Science and Technology, 50 Zhongling Street, Nanjing 210014, China; Jiangsu Co-Innovation Center for Prevention and Control of Important Animal Infectious Diseases and Zoonoses, 88 South Daxue Road, Yangzhou University, Yangzhou 225009, China; Institute of Veterinary Medicine, Jiangsu Academy of Agricultural Sciences, Key Laboratory of Veterinary Biological Engineering and Technology, Ministry of Agriculture; Jiangsu Key Laboratory for Food Quality and Safety-State Key Laboratory Cultivation Base of Ministry of Science and Technology, 50 Zhongling Street, Nanjing 210014, China; Jiangsu Co-Innovation Center for Prevention and Control of Important Animal Infectious Diseases and Zoonoses, 88 South Daxue Road, Yangzhou University, Yangzhou 225009, China; Institute of Veterinary Medicine, Jiangsu Academy of Agricultural Sciences, Key Laboratory of Veterinary Biological Engineering and Technology, Ministry of Agriculture; Jiangsu Key Laboratory for Food Quality and Safety-State Key Laboratory Cultivation Base of Ministry of Science and Technology, 50 Zhongling Street, Nanjing 210014, China; Jiangsu Co-Innovation Center for Prevention and Control of Important Animal Infectious Diseases and Zoonoses, 88 South Daxue Road, Yangzhou University, Yangzhou 225009, China; Institute of Veterinary Medicine, Jiangsu Academy of Agricultural Sciences, Key Laboratory of Veterinary Biological Engineering and Technology, Ministry of Agriculture; Jiangsu Key Laboratory for Food Quality and Safety-State Key Laboratory Cultivation Base of Ministry of Science and Technology, 50 Zhongling Street, Nanjing 210014, China; Jiangsu Co-Innovation Center for Prevention and Control of Important Animal Infectious Diseases and Zoonoses, 88 South Daxue Road, Yangzhou University, Yangzhou 225009, China; Institute of Veterinary Medicine, Jiangsu Academy of Agricultural Sciences, Key Laboratory of Veterinary Biological Engineering and Technology, Ministry of Agriculture; Jiangsu Key Laboratory for Food Quality and Safety-State Key Laboratory Cultivation Base of Ministry of Science and Technology, 50 Zhongling Street, Nanjing 210014, China; Jiangsu Co-Innovation Center for Prevention and Control of Important Animal Infectious Diseases and Zoonoses, 88 South Daxue Road, Yangzhou University, Yangzhou 225009, China; Institute of Veterinary Medicine, Jiangsu Academy of Agricultural Sciences, Key Laboratory of Veterinary Biological Engineering and Technology, Ministry of Agriculture; Jiangsu Key Laboratory for Food Quality and Safety-State Key Laboratory Cultivation Base of Ministry of Science and Technology, 50 Zhongling Street, Nanjing 210014, China; Jiangsu Co-Innovation Center for Prevention and Control of Important Animal Infectious Diseases and Zoonoses, 88 South Daxue Road, Yangzhou University, Yangzhou 225009, China; Institute of Veterinary Medicine, Jiangsu Academy of Agricultural Sciences, Key Laboratory of Veterinary Biological Engineering and Technology, Ministry of Agriculture; Jiangsu Key Laboratory for Food Quality and Safety-State Key Laboratory Cultivation Base of Ministry of Science and Technology, 50 Zhongling Street, Nanjing 210014, China; Jiangsu Co-Innovation Center for Prevention and Control of Important Animal Infectious Diseases and Zoonoses, 88 South Daxue Road, Yangzhou University, Yangzhou 225009, China; College of Veterinary Medicine, Nanjing Agricultural University, 1 Weigang, Xiaolingwei Street, Nanjing 210095, China; GuoTai (Taizhou) Center of Technology Innovation for Veterinary Biologicals, 28 Xinglin Road, Taizhou 225300, China; School of Food and Biological Engineering, Jiangsu University, 301 Xuefu Road, Xiangshan Street, Zhenjiang 212013, China

## Abstract

The precise role of the highly variable coronavirus S protein in modulating innate immune responses remains unclear. In this study, we demonstrated that the mutant strain of swine coronavirus porcine enteric diarrhea virus induced significantly lower levels of double-stranded RNA (dsRNA) accumulation, inhibited protein kinase R (PKR) activation and suppressed stress granule (SG) formation compared with the classical strain. The 29th amino acid at N-terminus of S was identified as the key functional site for regulation of SG formation, and found that mutant S inhibited PKR phosphorylation and SG formation by upregulating adenosine deaminase acting on RNA 1 (ADAR1)-p150. Notably, the Zα domain of ADAR1-p150 was essential for inhibiting SG formation. Upregulation of ADAR1-p150 also reduced accumulation of dsRNA depending on its RNA editing function. Virus rescue confirmed that the mutant carrying a substitution at amino acid 29 failed to induce ADAR1-p150, leading to dsRNA accumulation, PKR activation and SG formation. Interestingly, the latest severe acute respiratory syndrome coronavirus-2 strains exhibit a novel 25PPA27 deletion at N-terminus of S that was also shown to lead to altered ADAR1-p150 expression and SG inhibition. The transcription factor TCF7L2 was identified as a player in S-mediated transcriptional enhancement of ADAR1-p150. This study is the first to clarify the crucial role of N-terminus of S in immune regulation of coronaviruses.

## Graphical Abstract

**Graphical Abstract:**
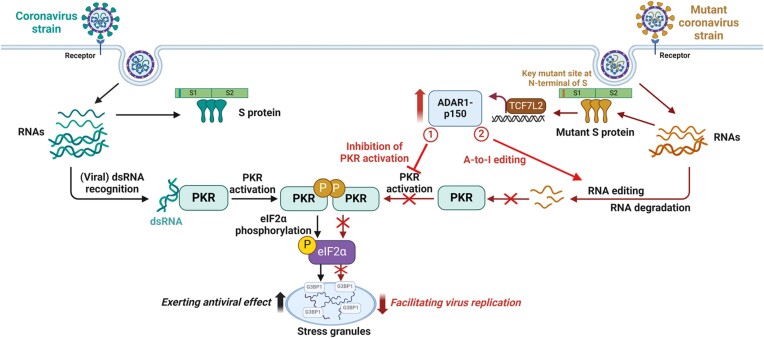

## Introduction

Coronaviruses belong to the Coronavirinae subfamily, encompassing four genera: alpha, beta, gamma and delta (1). As RNA viruses, they exhibit high mutation rates due to their replication mechanism and the absence of viral RNA polymerase proofreading activity (2). These mutations serve as building blocks of evolution, allowing for natural selection of traits that benefit the virus, such as immune evasion, enhanced virulence and adaptability (3). In the major pandemic outbreak of severe acute respiratory syndrome coronavirus-2 (SARS-CoV-2) in the human population, the virus evolved into alpha, beta, gamma, delta and omicron variants. Mutations in the viral nonstructural protein (nsp)1 have been found to suppress interferon I signaling (4), while mutations in open reading frame (ORF)8 have been suggested to augment viral transmission and immune evasion potential (5). Alphacoronaviruses and betacoronaviruses pose a major threat to livestock and include porcine transmissible gastroenteritis virus (TGEV), porcine enteric diarrhea virus (PEDV) and the recently emerged swine acute diarrhea syndrome coronavirus (SADS-CoV) (6), all of which cause intestinal syndromes in pigs. PEDV, initially discovered in 1971, is an ancient virus that particularly affects pigs (7). The classical PEDV strain gradually became endemic in European and Asian countries; however, since 2010, recurrent epidemics caused by highly virulent mutant strains of PEDV have emerged globally (8), resulting in 100% mortality in piglets aged 7 days or younger (9). The mutant PEDV strain exhibits novel immune-escape properties, such as mutations in nsp1 that inhibit C3 protein expression as a means to escape its antiviral effects (10). The coronavirus genome spans approximately 28 kb, with the 5′-terminus encompassing two-thirds of the genome and encoding the replicase polyproteins 1a and 1ab, which are cleaved into 16 nonstructural proteins, and the 3′ terminus encoding the spike (S), ORF3, envelope (E), membrane (M) and nucleocapsid (N) proteins. S is a glycoprotein that plays a crucial role in viral entry and determination of host cell tropism (11).

Stress granules (SGs) are dynamic cytosolic ribonucleoprotein (RNP) granules formed by mRNA- and other RNA-binding proteins in response to various environmental stimuli, including oxygen, thermal shock, nucleic acids and viral infections (12,13). Phosphorylation of the alpha subunit of eukaryotic initiation factor (eIF2α), which is induced by these stressors, leads to inhibition of cellular translation and consequently halts global protein synthesis, thereby significantly hindering viral replication (12). The double-stranded RNA (dsRNA) generated by RNA viruses upon cellular invasion is recognized by pattern recognition receptors (PRRs), triggering innate immune responses (14). Protein kinase R (PKR), activated by dsRNA, phosphorylates eIF2α at serine 51 (15,16). Although SGs can be activated independent of eIF2α, the PKR-eIF2α pathway is the primary route through which SG formation is triggered during viral infections (17).

Several viruses have developed strategies to avoid the formation or disassembly of antiviral SGs. Research on coronaviruses has shown that the Middle East respiratory syndrome coronavirus (MERS-CoV) accessory protein 4a restricts PKR activation by binding to dsRNA, thereby preventing the formation of SGs and promoting efficient viral protein translation (18,19). Infection with mouse hepatitis virus (MHV) results in host translational shutdown and mRNA degradation, leading to the formation of SGs and processing bodies (20). TGEV induces the formation of SG-like granules associated with viral transcription and replication (21). The N proteins of infectious bronchitis virus (IBV), SARS-CoV and SARS-CoV-2 interact with the essential SG component G3BP1 (22,23). Recent reports also show that IBV infection inhibits both eIF2α-dependent and -independent SG formation by reducing viral dsRNA levels and sequestering/depleting critical granule components via nsp15 (17,24). Induced SGs have been found to significantly inhibit PEDV replication (25). In addition, infection with some PEDV strains induces transient SG formation, with the virus subverting stable SG formation by inducing caspase-8 mediated G3BP1 cleavage (26) or downregulating G3BP1 expression via the viral papain-like protease (27). However, the differential induction of SGs by classical and mutant strains of PEDV and the underlying mechanisms remain unclear.

Adenosine deaminase acting on RNA 1 (ADAR1) is a dsRNA-binding protein and RNA-editing enzyme that catalyzes the C6 deamination of adenosine to produce inosine (A-to-I editing) in RNA substrates, including both host cellular and viral coding and noncoding RNAs (28,29). ADAR1 exists in two isoforms that differ only in the N-terminal region, with the p110 isoform being predominantly nuclear and the p150 isoform mostly localized in the cytoplasm (30). A-to-I editing by ADAR1 destabilizes duplex RNA, thereby inhibiting the activation of innate immunity, with most editing events attributed to the p150 isoform (31). ADAR1 also directly inhibits the activity of PKR, thereby preventing translational shutdown (32). Additionally, ADAR1 can function as a dsRNA-binding protein independently of its editing role, highlighting its multifunctionality with both editing-dependent and -independent activities (33).

In this study, classical and mutant PEDV strains were found to play different roles in SG induction in infected cells *in vitro*. The 29 amino acids at the N-terminus of S exhibited a critical role in ADAR1-p150 induction, subsequently affecting the accumulation of dsRNA, activation of PKR and formation of SGs. Additionally, the natural deletion of proline, proline and alanine at positions 25–27 (25PPA27) in the S protein of the latest prevalent SARS-CoV-2 strains also results in altered induction of ADAR1-p150. These findings provide initial evidence of the role of coronavirus S proteins in SGs regulation and highlight a novel immune evasion strategy adopted by mutant coronaviruses.

## Material and methods

### Cells and viruses

We used the African green monkey kidney epithelial cell lines Vero and Marc-145, intestinal porcine epithelial cell line IPEC-J2, and human hepatocarcinoma cell line Huh7, which were stored in our laboratory. All cell lines were cultured in Dulbecco’s modified Eagle’s medium (DMEM) supplemented with 10% (v/v) fetal calf serum. The classical PEDV strain JS2008 and the highly pathogenic mutant strain AH2012/12 were isolated from pig farms in China (34,35). Construction of the recombinant virus r12S-N29T was described previously (10). The tissue culture infectious dose 50 (TCID_50_) of each virus was determined by titration in Vero cells.

### Antibodies and reagents

The anti-dsRNA monoclonal antibody J2 was purchased from SCICONS (Szirák, Hungary). Rabbit polyclonal anti-G3BP1 (13057–2-AP), anti-G3BP2 (16276–1-AP), anti-PKR-like endoplasmic reticulum kinase (anti-PERK) (24390–1-AP), anti-CAPRIN1 (15112–1-AP), anti-ADAR1 (14330–1-AP) and anti-eIF3 (26178–1-AP) antibodies were purchased from Proteintech, Inc. (Wuhan, China). Rabbit monoclonal anti-ADAR1-p150 (A11466), anti-general control nonderepressible-2 (anti-GCN2) (A2307), anti-phospho-GCN2-T899 (AP1356) and anti-heme-regulated inhibitor (anti-HRI) (A24614) antibodies were purchased from ABclonal, Inc. (Wuhan, China). Mouse monoclonal anti-PEDV-N and anti-S antibodies were previously generated in the lab. Rabbit polyclonal anti-phospho-PERK (AF5902), rabbit polyclonal anti-eIF2α (AF6771), rabbit monoclonal anti-phospho-eIF2α (AF1237), rabbit monoclonal anti-PKR (AF2125) and rabbit monoclonal anti-phospho-PKR (AF1474) antibodies were purchased from Beyotime, Inc. (Shanghai, China). DyLight 647-conjugated goat anti-mouse immunoglobulin (Ig)G (BA1151), fluorescein isothiocyanate (FITC)-conjugated goat anti-mouse IgG (BA1101), FITC-conjugated goat anti-rabbit IgG (BA1105), Cy3-conjugated goat anti-mouse IgG (BA1031) and Cy3-conjugated goat anti-rabbit IgG (BA1032) were purchased from BOSTER, Inc. (Shanghai, China). Rabbit polyclonal anti-β-actin (A2066) and mouse monoclonal anti-Flag (F1804) antibodies, horseradish peroxidase (HRP)-conjugated goat anti-rabbit IgG (H + L) (AP307P) and goat anti-mouse IgG (H + L) (AP308P) antibodies, and sodium arsenite (SA, S7400) and cycloheximide (CHX, 239763-M) were purchased from Merck, Inc. (Darmstadt, Germany). 8-Azaadenosine (8-Azaad, HY-115686) was purchased from MCE, Inc. (Shanghai, China). Lipofectamine 3000 (L3000015) was purchased from Thermo Fisher Scientific, Inc. (MA, USA).

### Virus infections

Vero cells were cultured to form a confluent monolayer prior to exposure to AH2012/12, JS2008, rAH2012/12 or r12S-N29T at a multiplicity of infection (MOI) of 0.1 in the presence of 7.5 μg/ml trypsin. IPEC-J2 and Huh7 cells were incubated with PEDV strains at an MOI of 1 in the presence of 3 or 5 μg/ml trypsin, respectively. Following a 1-h incubation at 37°C to facilitate viral entry, cell samples were analyzed by western blotting, indirect immunofluorescence (IF), confocal microscopy and real-time quantitative PCR (qRT-PCR) at different time points post infection or treatment.

### qRT-PCR analysis

Total cellular RNAs were extracted using the FastPure Cell Total RNA Isolation Kit (Vazyme, Nanjing, China). The cDNAs were synthesized from 2 μg total RNA using the HiScript III 1st Strand cDNA Synthesis Kit (+gDNA wiper) (Vazyme), then used as template for quantitative PCR on a QuantStudio 6 real-time PCR system (Thermo Fisher) with AceQ Universal SYBR qPCR Master Mix (Vazyme). Specific primers (Supplementary Table S1) were used to detect the transcription levels of different genes.

### Plasmid construction and transfection

Except for nsp2, nsp3 and *S*, the amplicons of the non-structural and structural viral proteins were amplified from the cDNA of AH2012/12- or JS2008- infected cells and cloned into pcDNA3.1, which encodes a FLAG tag at the C-terminus of cloned proteins, using the recombinant cloning kit ClonExpress® II (Vazyme). The *nsp2*, *nsp3* and *S* genes of PEDV, as well as *S* of SARS-CoV-2 (GenBank No: NC045512), were synthesized by GenScript Biotech (Nanjing, China). The plasmid pmGFP-ADAR1-p150 (#117927) was purchased from Addgene (MA, USA), and the green fluorescent protein (GFP) was replicated with a His tag. Expression plasmids encoding mutant PEDV S, SARS-CoV-2 S and ADAR1-p150 proteins, and the ADAR1-p150 promoter reporter, were cloned using the Mut Express II Fast Mutagenesis Kit V2 (Vazyme). All primers are listed in Supplementary Table S1.

### Cell transfection

Cells were seeded on glass coverslips in 24-well plates and transfected with the specified plasmids and small interfering (si)RNA using Lipofectamine 3000 transfection reagent (Thermo Fisher Scientific), in accordance with the manufacturer’s recommendations. Briefly, plasmids were prepared by diluting 1.0 μg plasmid and 1.5 μl P3000 in 25 μl Opti-MEM (Thermo Fisher Scientific), while siRNA was prepared by diluting 100 nmol siRNA targeting ADAR1 or the negative control (siNC) in 25 μl Opti-MEM. For polyinosinic:polycytidylic acid (I:C), 5.0 μg/ml poly I:C was diluted and incubated in 25 μl Opti-MEM. After a 5-min incubation, the plasmid or siRNA mixtures were combined with Lipofectamine 3000 and incubated at room temperature for 10 min to form lipid-nucleic acid complexes, which were added to the cultured cells and incubated for required hours.

### IF and confocal microscopy

Cells were seeded on glass coverslips in 24-well plates to form monolayers. The cells were then subjected to plasmid transfection, viral infection or siRNA transfection. At the indicated time points, the cells were treated with SA (1 mM, 40 min), NaCl (200 mM, 50 min) or CHX (100 μg/ml, 1 h), poly I:C (5.0 μg/ml, 6 h), or the control reagents. Following treatment, the cells were fixed in ice-cold absolute ethanol for 10 min at 4°C. After three washes with phosphate-buffered saline (PBS), the cells were blocked with 3% bovine serum albumin in PBS for 1 h. The cells were then incubated with primary antibodies targeting G3BP1, G3BP2, eIF3, dsRNA, PEDV-N, PEDV-S, FLAG or His tag, diluted in blocking buffer, for 30 min at 37°C. This was followed by incubation with secondary antibodies conjugated with FITC, Cy3 or DyLight 647, diluted 1:300 in blocking buffer, for 20 min at 37°C. Nuclei were then stained with 4′,6′-diamidino-2-phenylindole (DAPI) for 5 min. Finally, cells were washed once with PBS and examined under a Zeiss LSM880 confocal microscope.

### Western blotting

Western blot analysis was performed as described previously, with a few modifications (10). Briefly, total cellular proteins were extracted from cells using different treatments. The obtained protein samples were separated by sodium dodecyl sulfate–polyacrylamide gel electrophoresis and transferred onto 0.22-μm polyvinylidene fluoride (PVDF) membranes (Millipore, Bedford, MA, USA). The membranes were blocked with 5% (w/v) nonfat milk overnight at 4°C and then probed with primary antibodies overnight at 4°C. After thorough washing with PBS + 0.1% (v/v) Tween 20 (PBST), the membranes were incubated with the corresponding HRP-conjugated secondary antibodies at 37°C for 1 h. The target proteins were developed with an enhanced chemiluminescence detection kit (Thermo Fisher Scientific), and images were taken using a Tanon™ 5200 CE Chemi-Image System (Tanon, Shanghai, China). The relative expression levels of target proteins were quantified using ImageJ software (version 1.8.0 for Windows) by comparing the grayscale intensity of β-actin or indicator cellular proteins, as previously described (10).

### Generation of recombinant viruses

Recombinant viruses containing mutant S were constructed using CRISPR/Cas9 technology following established protocols (36). Two single-guide RNAs targeting the S sequence were selected and synthesized *in vitro* via T7 transcription from templates amplified by overlapping PCR with forward primers sgRNAPEDV08IF/R and a constant reverse primer scaffold oligonucleotide. Then, the PEDV AH2012/12 genomic cDNA cloned into a bacterial artificial chromosome (BAC) plasmid was cleaved by Cas9 nuclease (NEB, Beijing, China) and purified. Subsequently, homologous recombination was carried out by co-incubating the purified BAC plasmid with DNA fragments containing the expected S mutations using an Infusion Clone Kit (TaKaRa, Dalian, China). Finally, 10 μl of the reaction mixture was transformed into DH10B cells (Biomed, Beijing, China) and the recombinant BAC plasmids were verified by sequencing. The primers used to construct the recombinant PEDVs are shown in Supplementary Table S1.

Confluent Vero cells were cultured in 6-well plates and transfected with recombinant BAC plasmids (6 μg/well) using Lipofectamine 3000. At 6 h post transfection, the cells were washed and supplemented with DMEM containing 7.5 μg/ml trypsin (2 ml/well). The cells were then placed in an incubator at 37°C and 5% CO_2_ to facilitate the recovery of infectious viruses. Daily microscopic observations were conducted to monitor the development of cytopathic effects. The characterization of recombinant viruses was carried out following established protocols (34).

### Luciferase assay

The ADAR1-p150 promoter reporter plasmid was constructed by cloning the –2000 to + 300 bp region around the transcription start site of exon 1A of *ADAR1*, as annotated in the Ensembl database, into the pGL3 basic vector. Exon 1A contains a methionine codon, which, upon translation initiation within exon 1A, leads to the synthesis of the p150 isoform (37). Vero cells were cultured in 24-well plates and transfected with a combination of wild-type (wt) S plasmids or mutants, the ADAR1-p150 luciferase reporter plasmid, and the control Renilla luciferase plasmid pRL-TK (Promega, Madison, WI, USA). At 36 h post transfection, the cells were lysed and transfectants were collected for analysis of firefly luciferase activity using a dual luciferase reporter assay system (Promega), following the manufacturer’s protocols. The results were then normalized to Renilla luciferase activity.

### Statistical analyses

Data are presented as means ± standard deviation (SD). Student’s *t*-tests and two-way analysis of variance tests were used to compare the data from different treatment groups. Differences with a *P*-value <0.05 were considered statistically significant. All statistical analyses and calculations were performed using GraphPad PRISM software (version 7.0 for Windows; GraphPad Software Inc.).

## Results

### Mutant PEDV infection leads to less dsRNA and inhibits SG formation in an eIF2α-dependent manner

**Figure 1.**
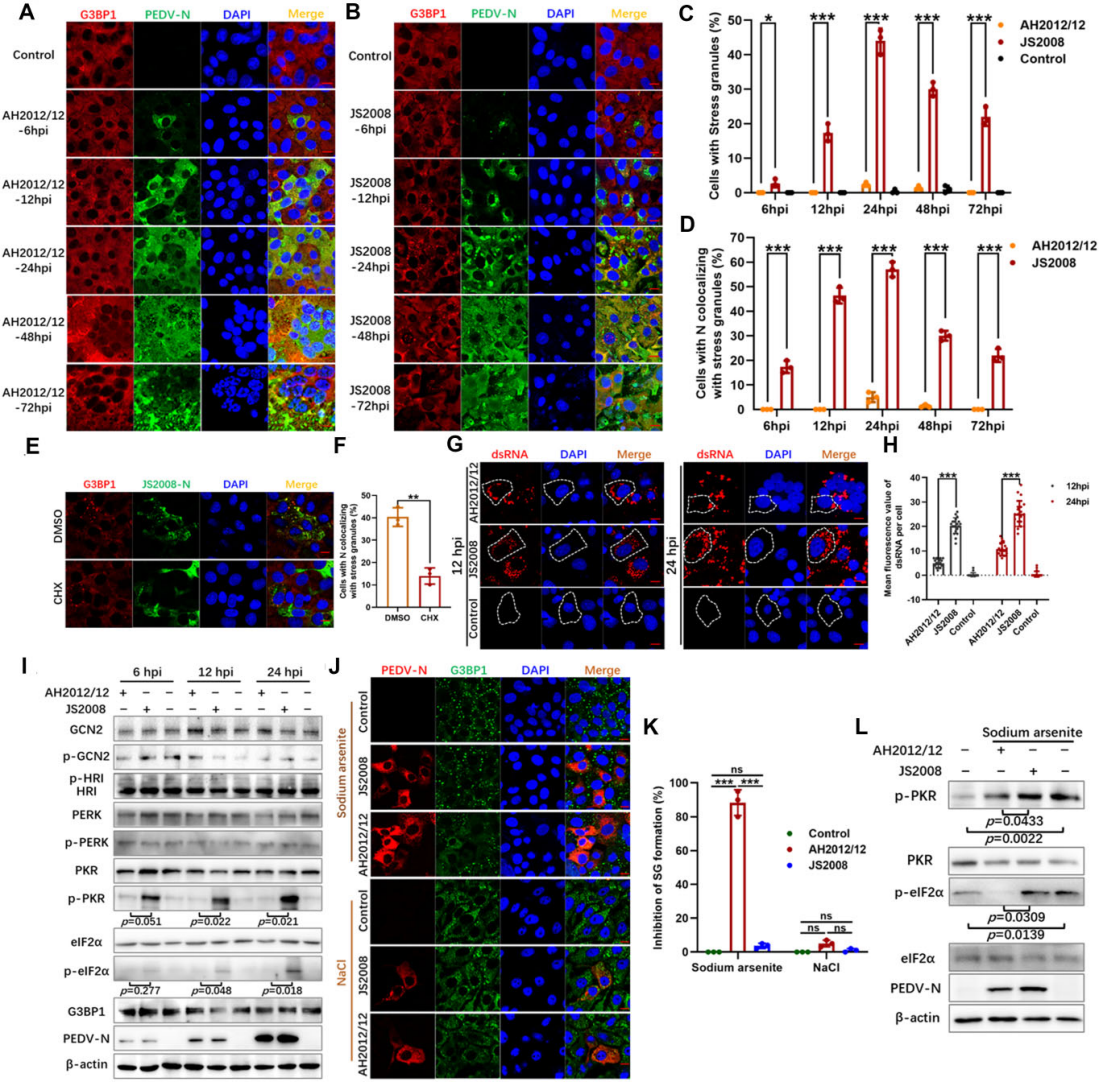
Compared with the classical strain, the mutant PEDV infection leads less dsRNA and inhibits SG formation in an eIF2α-dependent manner. Vero cells were infected with PEDV mutant strain AH2012/12 (**A**) or classical strain JS2008 (**B**) at an MOI of 0.1 or were mock infected. At the indicated time points, cells were fixed and incubated with anti-PEDV-N and anti-G3BP1 antibodies. The percentages of SGs-positive cells relative to all cells (**C**) and to the infected cells (**D**) were displayed in bar graphs. Vero cells were infected with JS2008 for 12 h, and the cells were treated with 100 μg/ml of CHX or with an equivalent volume of DMSO for 1 h. The cells were then immunostained with anti-G3BP1 and anti-PEDV-N antibodies (**E**and**F**). Vero cells were infected with AH2012/12 or JS2008, and were immunostained with dsRNA using the J2 antibody (**G**). The mean fluorescence values of dsRNA per cell were determined in 20 random cells (**H**). The PEDV infected cells were lysed to assay the expression levels of GCN2, p-GCN2, HRI, PERK, p-PERK, PKR, p-PKR, eIF2α, p-eIF2α, G3BP1 and PEDV-N using corresponding antibodies (**I**). Vero cells were infected with AH2012/12, JS2008 or were mock-infected. At 12 hpi, cells were treated with AS (1 mM, 40 min) (**K**) or NaCl (200 mM, 50 min), respectively (**J**). The cells were then fixed and incubated. The percentages of inhibition of SG formation represented the ratios of SGs negative cells to the virus infected cells, and were displayed in bar graphs (**K**). The infected cells were also detected for the expression levels of different proteins using corresponding antibodies (**L**). For (A–H), (J) and (K), the images are representative of three independent experiments. For (I) and (L), the figures are representative of two independent experiments. The relative expression levels of target proteins were determined by ImageJ according to the grayscale of β-actin in cells. The bar graphs showed the data of three independent experiments, presented as mean ± SD. Statistics: Student’s *t*-test or two-way ANOVA multiple comparisons tests (*P < 0.05, **P < 0.01, ***P < 0.001). Scale bars: 10 μm.

The continuous evolution of coronaviruses leads to the emergence of different strains with distinct immune characteristics. The classical strain and the high-virulence mutant strain that has been spreading since 2010 are the two most important strains of PEDV. However, the dynamics involved in SG formation and stress responses during infections with these two distinct strains remain unknown. We examined SG formation in Vero cells infected with the PEDV classical strain JS2008 and the mutant strain AH2012/12. As shown in Figure 1A and B, barely any SGs were detected in AH2012/12-infected cells throughout the infection period. However, in JS2008-infected cells, SGs were observed in up to 44% of cells at 24 h post infection (hpi), with this proportion decreasing over time. By the final stage of infection (72 hpi), the proportion of SG-positive cells decreased to 24% (Figure 1C). The kinetics of SG-positive cell accumulation in JS2008-infected cells were determined by counting the number of cells positive for both G3BP1 and N protein (SG-positive cells), as well as those positive for only N protein (SG-negative cells), at different time points, as described previously (18). At 6 hpi, only 17% of JS2008-infected cells were SG-positive. As the infection progressed, the percentage of SG-positive cells increased to 46% and 57% at 12 and 24 hpi, respectively. However, by the later stages of infection, the proportion of SG-positive cells had decreased to 22% (Figure 1D).

Detection of other SG markers, namely G3BP2 and eIF3, further identified the induction of canonical SG formation by JS2008 (Supplementary Figure S1A). To confirm that SGs and not RNP or RNA granules were formed in JS2008-infected Vero cells, we employed CHX treatment, which prevents SG formation by sequestering mRNAs on ribosomes (38,39). As shown in Figure 1E and F, compared with control dimethyl sulfoxide (DMSO), CHX treatment caused the dispersion of G3BP1-positive granules in JS2008-infected cells, supporting the authenticity of the SGs induced by JS2008 infection. PEDV infection also induced the formation of SGs in other cell types. As shown in Supplementary Figure S1B and C, in contrast to AH2012/12, typical SGs were apparent in 40% and 42% of Huh7 and IPEC-J2 cells infected with JS2008 at 24 hpi, respectively. These results indicated that the different SG induction patterns of JS2008 and AH2012/12 are consistent across different cell types.

Activation of the eIF2α kinase PKR by dsRNA triggers translational arrest and the formation of SGs. Therefore, we determined the levels of dsRNA in Vero cells infected with the two PEDV strains. IF analysis revealed a significantly higher accumulation of dsRNA in JS2008-infected Vero cells at 12 and 24 hpi compared with that in AH2012/12-infected cells (Figure 1G and H). Notably, dsRNA did not colocalize with G3BP1-positive granules, suggesting its exclusion from SGs (Supplementary Figure S1D).

There are three eIF2α kinases besides PKR that are involved in translation inhibition: GCN2, PERK and HRI (40). Therefore, we investigated the phosphorylation of PKR, GCN, PERK, HRI and eIF2α, and its potential impact on G3BP1 protein levels in Vero cells infected with the classical and mutant PEDV strains. The results revealed that, compared with the findings in uninfected control cells, there were no significant changes in the phosphorylation levels of PKR, GCN, PERK, HRI or eIF2α, or the protein levels of G3BP1 in AH2012/12-infected cells. By contrast, JS2008-infected cells exhibited significant increases in the phosphorylation levels of PKR and eIF2α (Figure 1I). However, the levels of phosphorylated PERK and total G3BP1 protein remained unchanged in JS2008-infected cells. These results implied that JS2008 induces SG formation by activating the PKR-eIF2α signaling pathway. The mutant PEDV strain AH2012/12 had no effect on this pathway, possibly because of its low production of dsRNA.

The inability of AH2012/12 to induce SG formation during infection suggested that it may have a role in suppressing SG formation. We sought to test this possibility using two stress-inducing stimuli: SA and sodium chloride (NaCl). SA triggers eIF2α phosphorylation in a PKR kinase dependent manner, leading to translational arrest and subsequent SG formation (41,42). Conversely, NaCl induces SGs in an eIF2α-independent manner by enhancing the local concentration of mRNAs and cellular proteins through cell volume reduction (43). In the absence of infection, both stimuli induced SG formation in >95% of Vero cells. Infection with the classical strain JS2008 had no effect on the SG formation induced by treatment with SA or NaCl. Interestingly, AH2012/12 infection inhibited ∼90% of the SA-induced SG formation in PEDV-N-positive cells, but did not significantly affect NaCl-induced SG formation (Figure 1J and K). Next, we used western blotting to assess the AH2012/12-mediated inhibition of eIF2α phosphorylation. As shown in Figure 1L, compared with JS2008-infected and uninfected control cells under treatment with SA, AH2012/12-infected cells had significantly decreased phosphorylation levels of PKR and eIF2α. These results indicated that the mutant PEDV strain AH2012/12 inhibits SG formation by inhibiting the phosphorylation of eIF2α.

### S protein of mutant PEDV significantly inhibits SG formation

**Figure 2.**
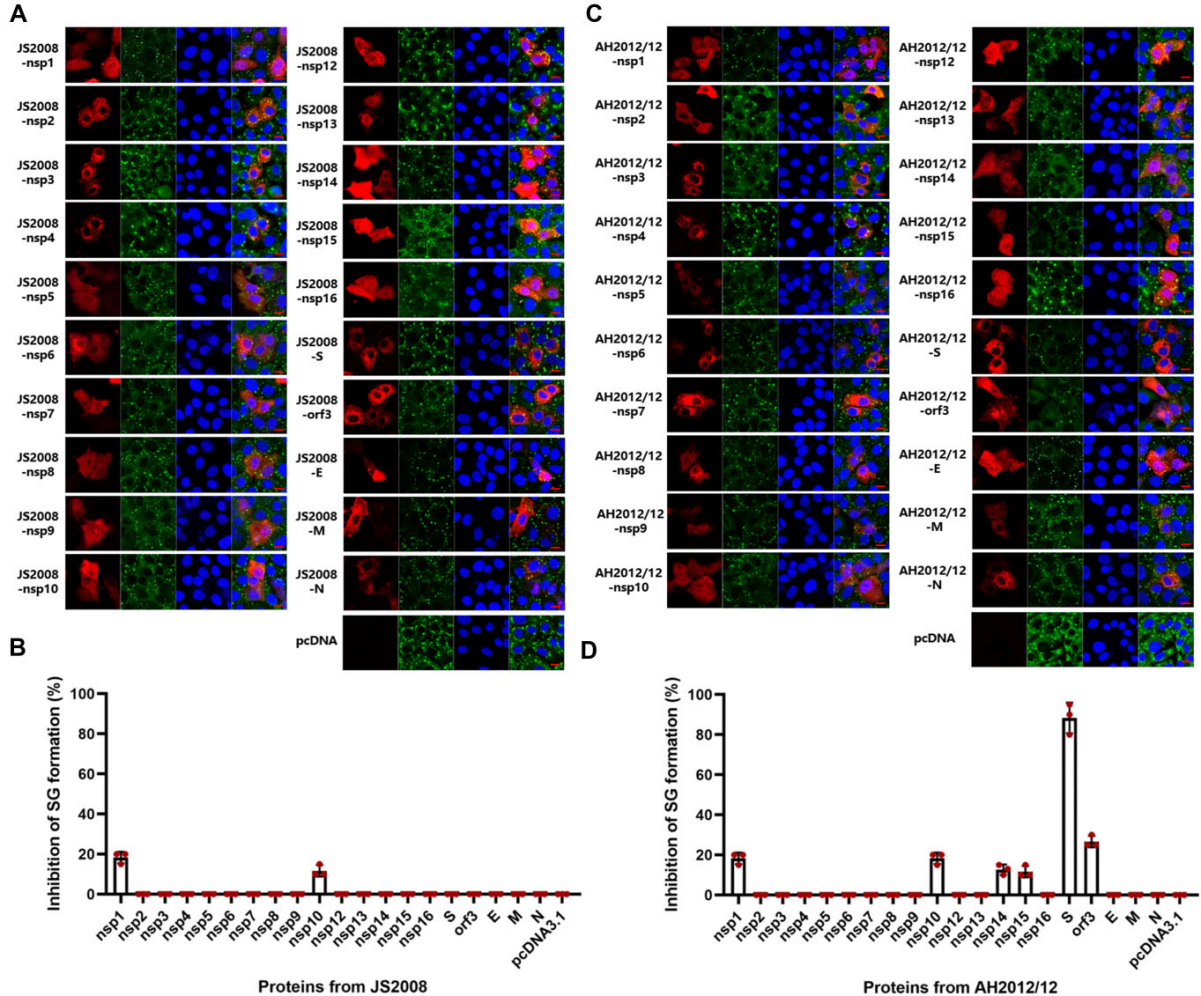
Screening viral proteins that inhibit SG formation. Vero cells were transfected with plasmids encoding Flag-tagged proteins of JS2008 (**A**) or AH2012/12 (**C**). At 24 h post-transfection, cells were treated with AS. The cells were fixed and incubated with anti-Flag and anti-G3BP1 antibodies. Images are representative of three independent experiments. The percentages of inhibition of SG formation relative to JS2008 (**B**) or AH2012/12 (**D**) protein expressing cells from three independent experiments were shown in the bar graphs, presented as the mean ± SD. Scale bars: 10 μm.

To identify the viral proteins involved in the inhibition of SG formation, each of the structural and nonstructural proteins of AH2012/12 and JS2008 were transfected into Vero cells treated with SA to trigger the formation of SGs. As shown in Figure 2A and B, nsp1 and nsp10 from the classical strain JS2008 inhibited SG formation by 18% and 12%, respectively. The S protein from mutant AH2012/12 substantially inhibited SG formation by 88% (Figure 2C and D), while nsp1, nsp10, nsp14, nsp15 and orf3 from AH2012/12 also inhibited SG formation by 18%, 18%, 13%, 11% and 27%, respectively. These results indicated that the mutant strain carries viral proteins that are more potent in the inhibition of SG formation. Among these, the S protein from AH2012/12 exhibited the most outstanding capacity for SGs suppression.

### Amino acid 29 of mutant PEDV S protein is critical for inhibiting SG formation

**Figure 3.**
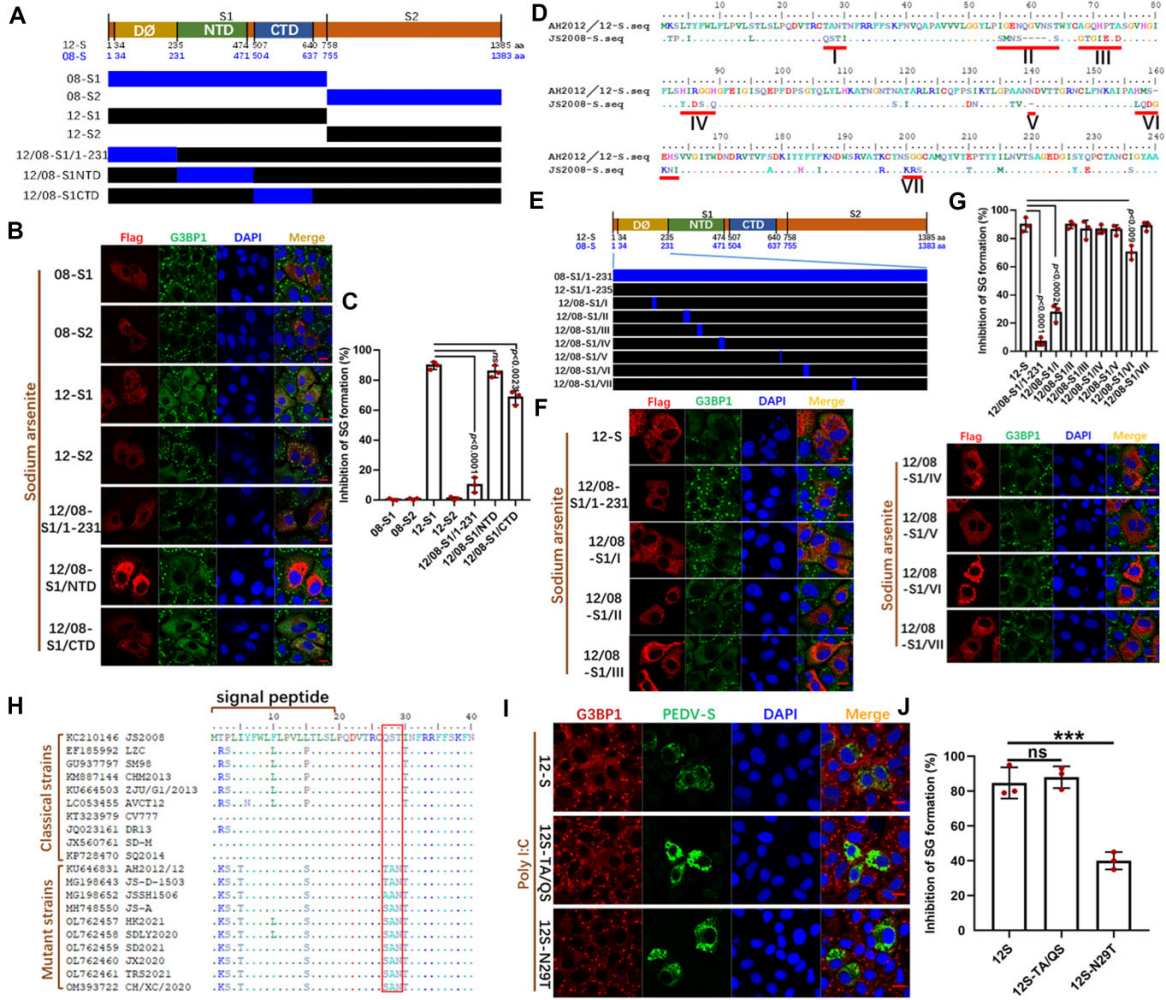
The 29th amino acid of the mutant PEDV S protein is critical for inhibiting SGs formation. (**A**) Schematic diagram of the structural composition of AH2012/12 S (12-S) and JS2008 S (08-S), and the constructions of S mutants. Vero cells were transfected with recombinant plasmids of mutant S proteins (**B**). At 24 h post-transfection, cells were treated with AS, and then fixed and incubated with anti-Flag antibody and anti-G3BP1. The percentages of inhibition of SG formation relative to cells expressing mutant S proteins were displayed in the bar graph (**C**). Amino acid sequence alignment of the N-terminal of AH2012/12 and JS2008 S proteins revealed I-VII mutations (**D**). Schematic diagram of the constructions of mutant S proteins with I-VII mutations (**E**). Vero cells were transfected with I-VII mutant S expressing plasmids (**F**). At 24 h post-transfection, cells were treated with AS and the SGs formation and proteins expression were determined. The percentages of inhibition of SG formation relative to cells expressing I-VII mutant S proteins were displayed in the bar graph (**G**). Amino acid sequence alignment of the N-terminal of PEDV mutant and classical strains (**H**). Vero cells were transfected with 12-S, 12S-TA/QS and 12S-N29T-expressing plasmids (**I**). At 18 h post-transfection, cells were transfected with poly I:C for 6 h, and then the SG formation and proteins expression were determined. The percentages of inhibition of SG formation relative to cell expressing S were displayed in the bar graph (**J**). For (B), (F) and (I), the images are representative of three independent experiments. For (C), (G) and (J), the bar graphs showed the data of three independent experiments, presented as mean ± SD. Statistics: Student’s *t*-test (***P< 0.001). Scale bars: 10 μm.

To investigate the specific region of S protein involved in regulating SG formation, we constructed plasmids that express the S1 and S2 proteins, which derived from AH2012/12 and JS2008 (Figure 3A). Neither the S1 nor S2 protein from JS2008 inhibited SG formation induced by SA in Vero cells. In contrast, the S1 protein from AH2012/12 was able to block SG formation, whereas the S2 protein from AH2012/12 was not (Figure 3B and C).

Next, the different structural domains of the domain 0 (DØ), N-terminal domain (NTD) and C-terminal domain (CTD) from the AH2012/12 S1 protein were substituted with those from the JS2008 S1 protein to generate recombinant expression plasmids. As shown in Figure 3B and C, the recombinant S protein 12/08-S1/1–231 exhibited a significant loss in the ability to inhibit SG formation, dropping from 90% to only 10% inhibition. Conversely, the inhibitory effect of the 12/08-S1/NTD on SG formation remained unchanged, while that of the 12/08-S1/CTD exhibited a certain inhibition of SG formation (Figure 3B and C). These results indicated that amino acids 1–231 of the mutant S protein are crucial for regulating SG formation.

To clarify the key sites in the S protein that regulate SG formation, we performed a sequence alignment of amino acids 1–240 from the two strains, which revealed multiple discrepancies, including seven consecutive amino acid mutations or deletions (Figure 3D). Recombinant expression vectors were then constructed to replace these seven positions (I–VII) from strain 12-S1 with those from strain 08-S (Figure 3E). In IF assays of SA-induced Vero cells, the mutant 12/08-S1/I exhibited a significantly reduced inhibitory effect on SG formation, dropping from 90% to 27% (Figure 3F and G). The mutant 12/08-S1/VI retained some inhibitory ability, which decreased from 90% to 70% inhibition (Figure 3G). These results revealed that amino acids 27–30 of the S protein are critical for regulating SG formation. Comparison of these amino acids in the classical and mutant strains revealed the 27T/SAN29 as the main mutation sites, with the 28th and 29th amino acids being highly conserved (Figure 3H). Next, we constructed the recombinant plasmids 12S-TA/QS and 12S-N29T. Compared with 12-S, 12S-N29T exhibited a significantly decreased ability to inhibit SA-induced SG formation (Supplementary Figure S2). Because SA can induce SG formation in PKR-deficient cells (44), we employed poly I:C, which activates PKR-mediated eIF2α phosphorylation, to stimulate SG formation in Vero cells (45). As shown in Figure 3I and J, 12-S was able to significantly inhibit SG formation induced by poly I:C, with 12S-N29T showing a reduced inhibitory effect. These findings highlighted the crucial role of the 29th amino acid, N, in mutant PEDV S protein in the inhibition of SG formation through the PKR-eIF2α pathway.

### Mutant S protein inhibits PKR phosphorylation and SG formation by upregulating ADAR1-p150 expression, with the Zα domain playing a crucial role

**Figure 4.**
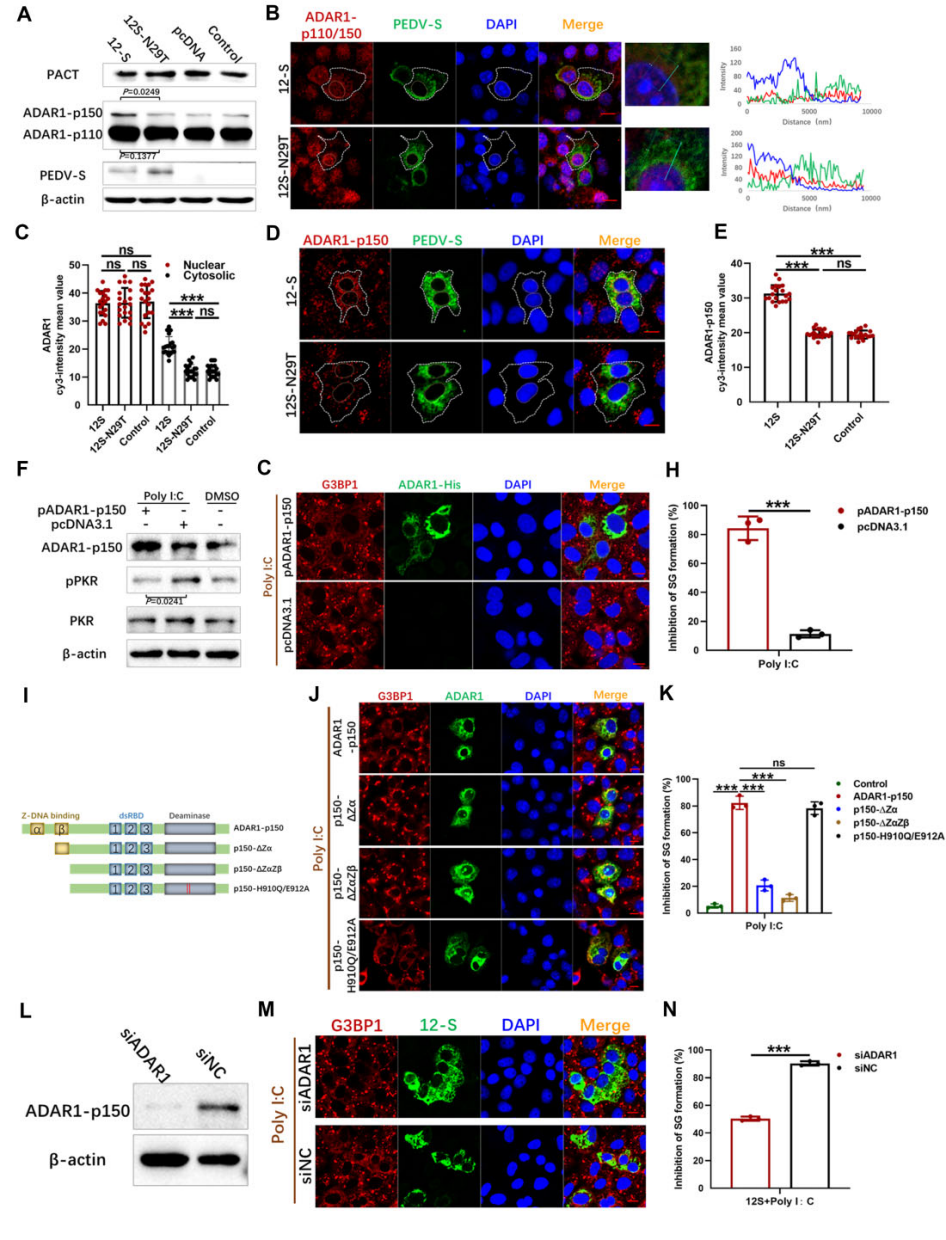
The S protein of mutant PEDV inhibits PKR phosphorylation and SG formation by upregulating ADAR1-p150 expression, and the Zα domain plays a crucial role in exerting inhibitory effects. Vero cells were transfected with 12-S or 12S-N29T. At 24 h post-transfection, the expression levels of PACT, ADAR1, PEDV-S and β-actin were detected (**A**). The transfected cells were also fixed and incubated with anti-PEDV-S and anti-ADAR1-p150/110 or anti-ADAR1-p150 (**B** and **D**), and the mean fluorescence values of ADAR1 per cell were determined in 20 random cells (**C** and**E**). Vero cells were transfected with the recombinant plasmid pADAR1-p150. At 18 h post-transfection, the cells were transfected with poly I:C for 6 h, and then the expression levels of ADAR1-p150, PKR, pPKR and β-actin were detected (**F**). The cells were also fixed and incubated with an anti-His antibody and an anti-G3BP1 antibody (**G**). The percentages of inhibition of SG formation relative to ADAR1-p150 expressing cells were displayed in the bar graph (**H**). (**I**) A schematic diagram of the structural composition of ADAR1-p150 and the constructed mutants is shown. Vero cells were transfected with mutant ADAR1-p150 plasmids. At 18 h post-transfection, the cells were transfected with poly I:C for 6 h, then fixed and incubated with anti-His and anti-G3BP1 antibodies (**J**). The percentages of inhibition of SG formation relative to mutant ADAR1-p150 expressing cells were displayed in the bar graph (**K**). Vero cells were transfected with siADAR1 or siNC for 24 h, then all cells were transfected with 12-S. At another 18 h post-transfection, cells were transfected with poly I:C for 6 h, then the expression of ADAR1-p150 was detected by western blot (**L**). Fixed cells were incubated with anti-PEDV-S and anti-G3BP1 antibodies (**M**). The percentages of inhibition of SG formation relative to 12-S expressing cells were displayed in the bar graph (**N**). For (A) and (F), the figures are representative of two independent experiments. For (B), (D), (G), (J) and (M), the images are representative of three independent experiments. The relative expression levels of target proteins according to the grayscale of β-actin in cells were also determined by ImageJ. The bar graphs of (H), (K) and (N) showed the data of three independent experiments, presented as mean ± SD. Statistics: Student’s *t*-test (***P < 0.001). Scale bars: 10 μm.

The mutant PEDV S protein inhibited SG formation induced by poly I:C, which specifically activates PKR-mediated eIF2α phosphorylation. Common strategies to impede SG formation include disrupting PKR and eIF2α activation sites and blocking interactions among key SG components (46). The immunoprecipitation assay of potential interactions between the mutant S and PKR, eIF2α, G3BP1 and CAPRIN1 proteins did not detect any interactions (Supplementary Figure S3). Thus, we hypothesized that intermediary proteins may disrupt the SG formation pathway. ADAR1 is known to directly inhibit PKR activity (32), while the PKR activator (PACT) activates PKR under cellular stress (41,42). Next, we sought to examine the effects of 12-S and 12S-N29T proteins on ADAR1 and PACT expression. As shown in Figure 4A, overexpression of 12-S did not affect the expression of PACT but significantly upregulated the levels of the ADAR1 p150 isoform, without a notable change in the p110 isoform. Considering the different intracellular localization patterns of the p150 and p110 isoforms, we employed IF with p150/110-specific antibodies. This revealed a significant upregulation of cytoplasmic ADAR1 expression upon 12-S overexpression, with nuclear ADAR1 expression remaining unaffected. Conversely, the N29T mutant S protein did not alter the expression of ADAR1 in either the cytoplasm or the nucleus (Figure 4B and C). Furthermore, IF employing p150-specific antibodies demonstrated that overexpression of the wt S protein amplified the cytoplasmic localization of ADAR1-p150, while the N29T mutant S protein had no effect on ADAR1-p150 expression (Figure 4D and E).

The role of ADAR1-p150 in inhibiting SG formation was clarified through overexpression of ADAR1-p150, which significantly inhibited PKR phosphorylation and SG formation in response to poly I:C treatment (Figure 4F–H). To determine the domain of ADAR1-p150 responsible for inhibiting SG formation, mutant variants of ADAR1-p150 were generated by deleting the Z-DNA-binding domains, Zα or Zα/β, and by introducing point mutations (H910Q and E912A) to disrupt the catalytic CHAE motif and thus the C-terminal deaminase activity, as previously outlined (44,47) (Figure 4I). Deletion of the Zα domain was found to abolish the inhibition of SG formation (Figure 4J and K). These findings demonstrated that the Zα RNA-binding domain of ADAR1-p150 is crucial for inhibiting the formation of SGs, indicating that this function is separate from its deaminase activity.

The inhibitory effect of 12-S protein on SG formation through upregulation of ADAR1-p150 was further examined using siRNA. As shown in Figure 4L–N, the inhibitory effect of 12-S on poly I:C-induced SG formation was significantly reduced by knockdown of ADAR1 through transfection of ADAR1-specific siRNA. These data suggested that the S protein regulates the formation of SGs by modulating the expression of ADAR1-p150, with amino acid 29 serving as a critical regulatory site.

### S protein-induced ADAR1-p150 inhibits the accumulation of dsRNA, with its deaminase activity playing a pivotal role

**Figure 5.**
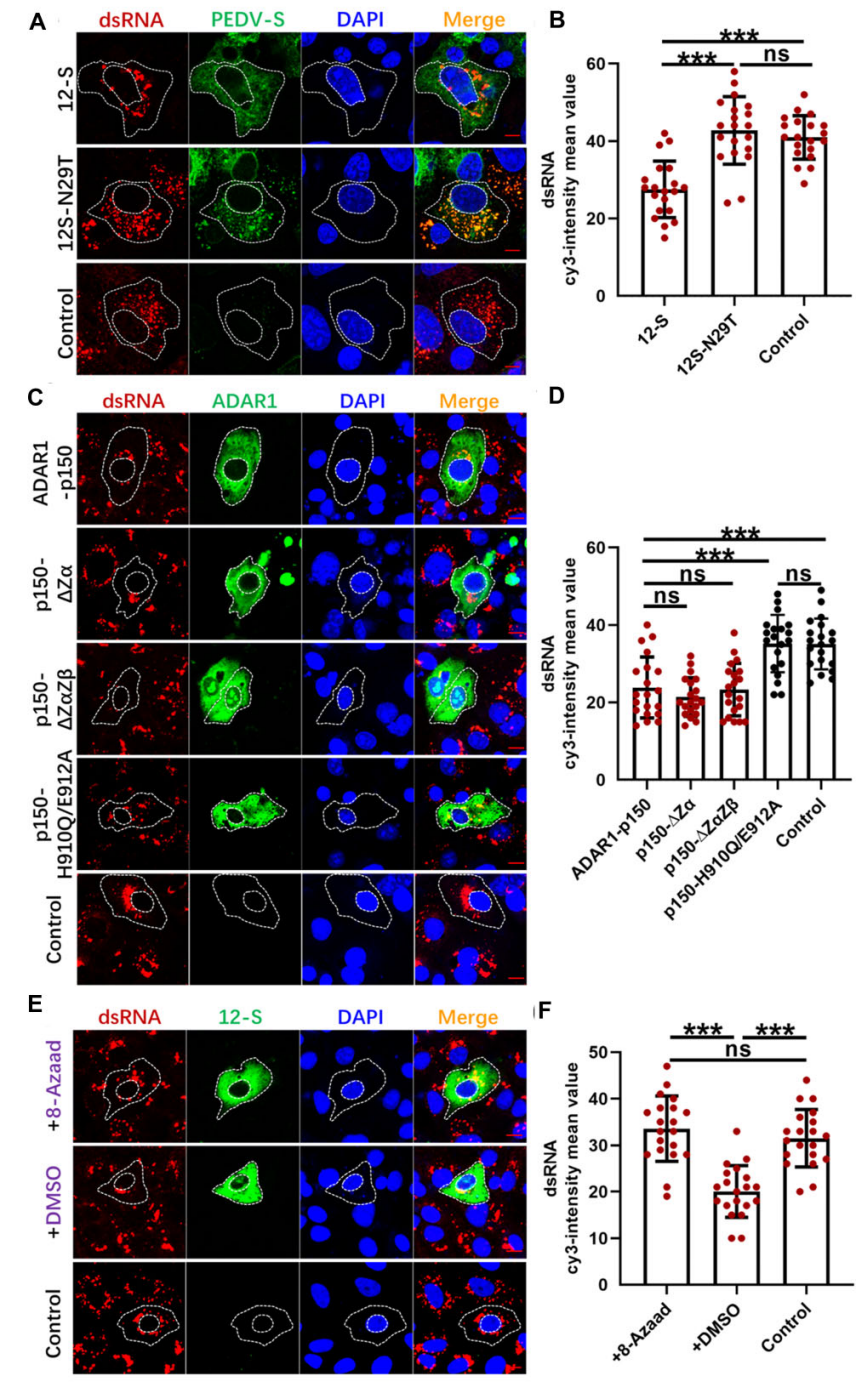
The ADAR1-p150 induced by S protein inhibits the accumulation of dsRNA, with its deaminase activity playing a pivotal role in this process. Vero cells were transfected with recombinant plasmids 12-S or 12S-N29T. At 24 h post-transfection, cells were infected with JS2008. At 12 h post-infection, cells were fixed and incubated with anti-PEDV-S antibody and anti-dsRNA antibodies (**A**), and the mean fluorescence values of dsRNA per cell were determined in 20 random cells (**B**). Vero cells were transfected with mutant ADAR1-p150 plasmids. At 24 h post-transfection, cells were infected with JS2008. At 16 h post-infection, cells were fixed and incubated with anti-His antibody and anti-dsRNA antibodies (**C**), and the mean fluorescence values of dsRNA per cell were determined in 20 random cells (**D)**. Vero cells were transfected with mutant 12-S plasmid. At 24 h post-transfection, cells were infected with JS2008 and simultaneously treated with 8-Azaad or DMSO. At 16 h post-infection, cells were fixed and incubated with anti-PEDV-S antibody and anti-dsRNA antibodies (**E**), and the mean fluorescence values of dsRNA per cell were determined in 20 random cells (**F**). For (A), (C) and (E), the images are representative of three independent experiments. Data are shown as the mean ± SD. Statistics: Student’s *t*-test (***P < 0.001). Scale bars: 10 μm.

Our findings in Vero cells indicated that JS2008 infection leads to higher dsRNA accumulation than AH2012/12 infection, with the AH2012/12 S protein upregulating ADAR1-p150 expression. Thus, we hypothesized that the mutant S protein suppresses dsRNA generation by modulating the expression of ADAR1-p150. The influence of both 12-S and its variant 12S-N29T on dsRNA production in JS2008-infected Vero cells was explored. The results showed that 12-S expression notably diminished the production of viral dsRNA, whereas 12S-N29T had no effect (Figure 5A and B). Next, the influence of ADAR1-p150 mutants on dsRNA production was examined. As shown in Figure 5C and D, compared with the control group, overexpression of both wt ADAR1-p150 and mutant ADAR1-p150 proteins lacking the Zα and Zβ DNA-binding domains significantly reduced the dsRNA content following JS2008 infection. However, in the absence of its deaminase activity, ADAR1-p150 had no effect on the dsRNA produced by JS2008 infection, indicating that the A-I editing activity of ADAR1-p150 is key to its downregulation of dsRNA accumulation. To further clarify the role of 12-S in reducing dsRNA via upregulation of ADAR1-p150, Vero cells were inoculated with JS2008 after overexpression of 12-S. The results showed that, compared with control DMSO, treatment with 8-Azaad, a specific inhibitor of ADAR1 editing activity, significantly weakened the inhibitory effect of 12-S on dsRNA accumulation, bringing the dsRNA content closer to that of normal virus-infected cells (Figure 5E and F). These data suggested that the S protein of mutant PEDV downregulates the content of cytoplasmic dsRNA by inducing ADAR1-p150.

### Viral mutant strain r12S-N29T exhibits loss of ADAR1-p150 induction and increased dsRNA and SG production

**Figure 6.**
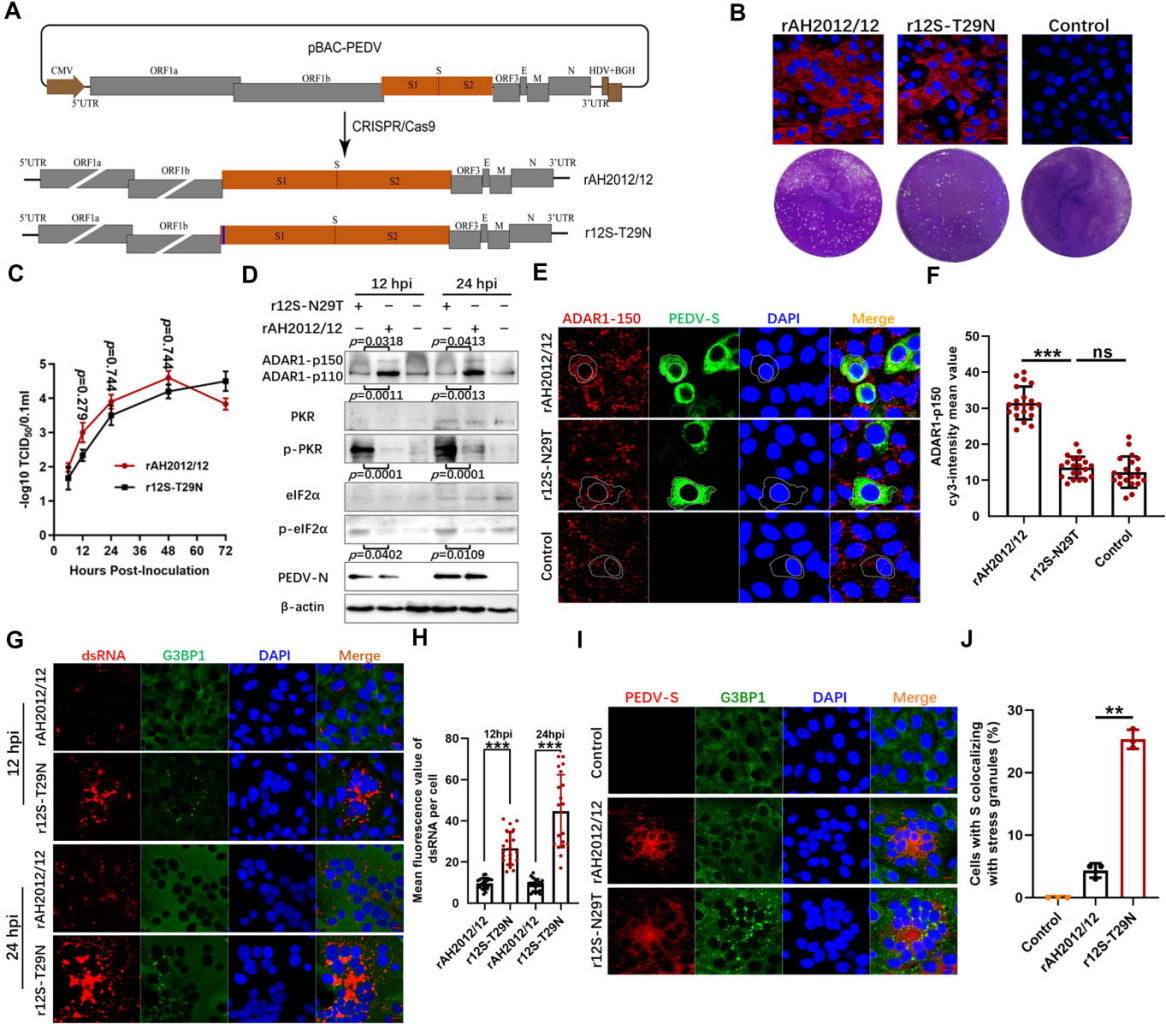
The S-N29T mutant strain loses ADAR1-p150 induction and produces more dsRNA and SGs. Construction of recombinant PEDV strain with the 29th amino acid mutation. (**A**) Schematic diagram of the structural composition of recombinant virus rAH2012/12 and r12S-T29N. Vero cells were infected with the mutants, and the specific fluorescence of N protein was detected using IFs at 24 hpi. The plaques of cells infected with the viruses were observed at 3 days post infection (**B**). The growth curves of viruses in Vero cells were determined by measuring the virus titers at different time points after viral infection (**C**), and the dot graph showed the data of three independent determinations, presented as the mean ± SD. Vero cells were infected with rAH2012/12 or r12-N29T at 0.1 MOI for 12 and 24 h. The infected cells were detected for the expression levels of ADAR1, PKR, p-PKR, eIF2α, p-eIF2α, PEDV-N and β-actin (**D**). Figures are representative of two independent experiments. The relative expression levels of target proteins ADAR1, p-PKR and p-eIF2α were determined by measuring grayscale of β-actin in cells using ImageJ. The cells infected for 12 h were immunostained with ADAR1-p150 and PEDV-S (**E**), and the mean fluorescence values of ADAR1-p150 per cell were determined in 20 random cells (**F**). The cells infected for 12 and 24 h were immunostained with anti-dsRNA and anti-G3BP1 antibodies (**G**), and the mean fluorescence values of dsRNA per cell were determined in 20 random cells (**H**). The cells infected for 24 h were immunostained with anti-G3BP1 and anti-PEDV-S antibodies (**I**). The percentages of SGs-positive cells relative to infected cells of three independent experiments were displayed in bar graphs (**J**). For (E), (G) and (I), the images are representative of three independent experiments. Data are shown as the mean ± SD. Statistics: Student’s *t*-test (**P < 0.01, ***P < 0.001). Scale bars: 10 μm.

To verify the regulatory effect of PEDV S protein on ADAR1-p150 at the viral particle level, we constructed the mutant strain r12S-N29T by introducing the N29T mutation from the classical strain S protein into the backbone of the mutant strain AH2012/12 (Figure 6A). Biological assays showed that the mutant strain infected Vero cells with similar effectiveness to the parental strain (Figure 6B). Compared with rAH2012/12, the replication kinetics of r12S-N29T showed a slight decrease, as indicated by plaque assays and replication curves (Figure 6B and C). Infection of Vero cells with the recombinant strains revealed that r12S-N29T lost the ability to upregulate the expression of ADAR1-p150, and instead activated the PKR-eIF2α pathway (Figure 6D). IF assays further confirmed that rAH2012/12 infection upregulated the expression of ADAR1-p150 in cytoplasm, while r12S-N29T lost this capacity (Figure 6E and F).

As shown above, JS2008-infected Vero cells accumulated more dsRNA than AH2012/12-infected cells. At 12 and 24 hpi, the intracellular dsRNA production was significantly higher in r12S-T29N-infected cells than in rAH2012/12-infected cells (Figure 6G and H). The presence of this dsRNA further activates the PKR-eIF2α pathway, causing translation stagnation and leading to SG production. Examination of the SGs induced by the recombinant strains revealed that, at 24 hpi, only ∼5% of rAH2012/12-infected cells were SG-positive (Figure 6I and J), whereas infection with r12S-T29N significantly increased SG induction, with 23% of infected cells showing positivity for SGs.

### The 25PPA27 deletion mutant of SARS-CoV-2 S protein exhibits loss of ADAR1-p150 induction

**Figure 7.**
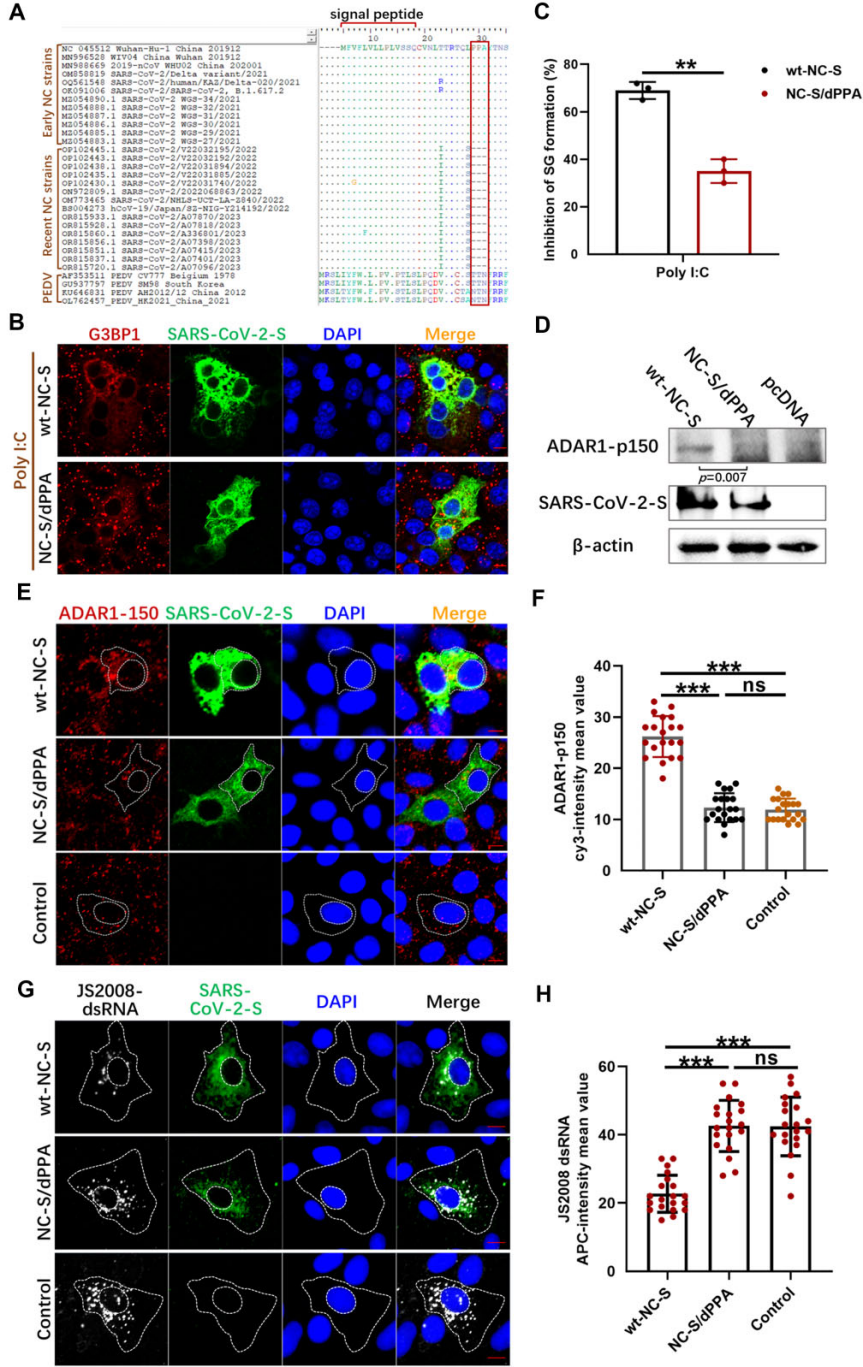
The 25PPA27 deletion of SARS-CoV-2 S protein loses ADAR1-p150 induction. Amino acid sequence alignment of the N-terminals of SARS-CoV-2 early and recent strains (**A**). Vero cells were transfected with plasmids expressing wt-NC-S or NC-S/dPPA. At 18 h post-transfection, cells were transfected with poly I:C and immunostained with anti-G3BP1 and anti-SARS-CoV-2-S antibodies (**B**). The percentages of SGs-positive cells relative to transfected cells of three independent experiments were displayed in bar graphs (**C**). Vero cells transfected with wt-NC-S or NC-S/dPPA were analyzed for the expression levels of ADAR1-p150, SARS-CoV-2-S and β-actin using corresponding antibodies at 24 h post-transfection (**D**). The relative expression levels of target proteins ADAR1-p150 according to the grayscale of β-actin in cells were also determined using ImageJ. The transfected cells were also fixed, incubated with anti-SARS-CoV-2-S and anti-ADAR1-p150 antibodies (**E**), and the mean fluorescence values of ADAR1-p150 per cell were determined in 20 random cells (**F**). Vero cells were transfected with wt-NC-S or NC-S/dPPA. At 24 h post-transfection, cells were infected with JS2008. At 12 h post-infection, cells were fixed and incubated with anti-PEDV-S antibody and anti-dsRNA antibodies (**G**), and the mean fluorescence values of dsRNA per cell were determined in 20 random cells (**H**). For (B), (E) and (G), the images are representative of three independent experiments. Data are shown as the mean ± SD. Statistics: Student’s *t*-test (**P<0.01, ***P<0.001). Scale bars: 10 μm.

The N-terminal region of the PEDV S protein displays notable variability across different strain subtypes (48). This study identified a high mutational frequency in amino acids 27–29, which lie adjacent to the signal region of the S protein, impacting the expression of ADAR1-p150 and the accumulation of dsRNA. To investigate similar characteristics in other coronaviruses, the N-terminal sequences of the S proteins from the alphacoronavirus TGEV, the betacoronavirus SARS-CoV-2, and the deltacoronavirus porcine deltacoronavirus (PDCoV) were further analyzed. We found no significant mutations in the N-terminal regions of TGEV and PDCoV S proteins (Supplementary Figure S4). By contrast, the recent epidemic Omicron strains of SARS-CoV-2 exhibited a deletion at amino acids 25–27 compared with the original Wuhan and Delta strains (Figure 7A). Therefore, a mutant expression plasmid lacking the 25PPA27 deletion (NC-S/dPPA) was constructed using the wt-NC-S recombinant expression plasmid as a template.

First, we confirmed the inhibitory effect of SARS-CoV-2 S protein on SG formation in Vero cells. As shown in Figure 7B and C, transfection of the SARS-CoV-2 S expression plasmid induced cell fusion, as described previously (49,50). While the expression of wt-NC-S also significantly inhibited the formation of poly I:C-induced SGs, NC-S/dPPA lost this inhibitory ability. Next, we examined the effect of SARS-CoV-2 S protein on ADAR1-p150 expression. Overexpression experiments demonstrated that wt-NC-S upregulated ADAR1-p150 expression, whereas NC-S/dPPA did not (Figure 7D). IF assays further showed that, compared with the control, wt-NC-S significantly enhanced the fluorescence intensity of cytoplasmic ADAR1-p150, whereas NC-S/dPPA had no effect (Figure 7E and F). Exploration of the influence of these S proteins on dsRNA production in JS2008-infected cells showed that wt-NC-S notably diminished the production of viral dsRNA. In contrast, the expression of NC-S/dPPA appeared to have no effect on dsRNA production compared with the control (Figure 7G and H). These results demonstrated that the deletion of amino acids 25–27 in the N-terminal region of SARS-CoV-2 S protein alters the expression of ADAR1-p150, influencing the inhibition of SG formation and the accumulation of dsRNA.

### S proteins of PEDV and SARS-CoV-2 transcriptionally promote ADAR1-p150 expression via TCF7L2

**Figure 8.**
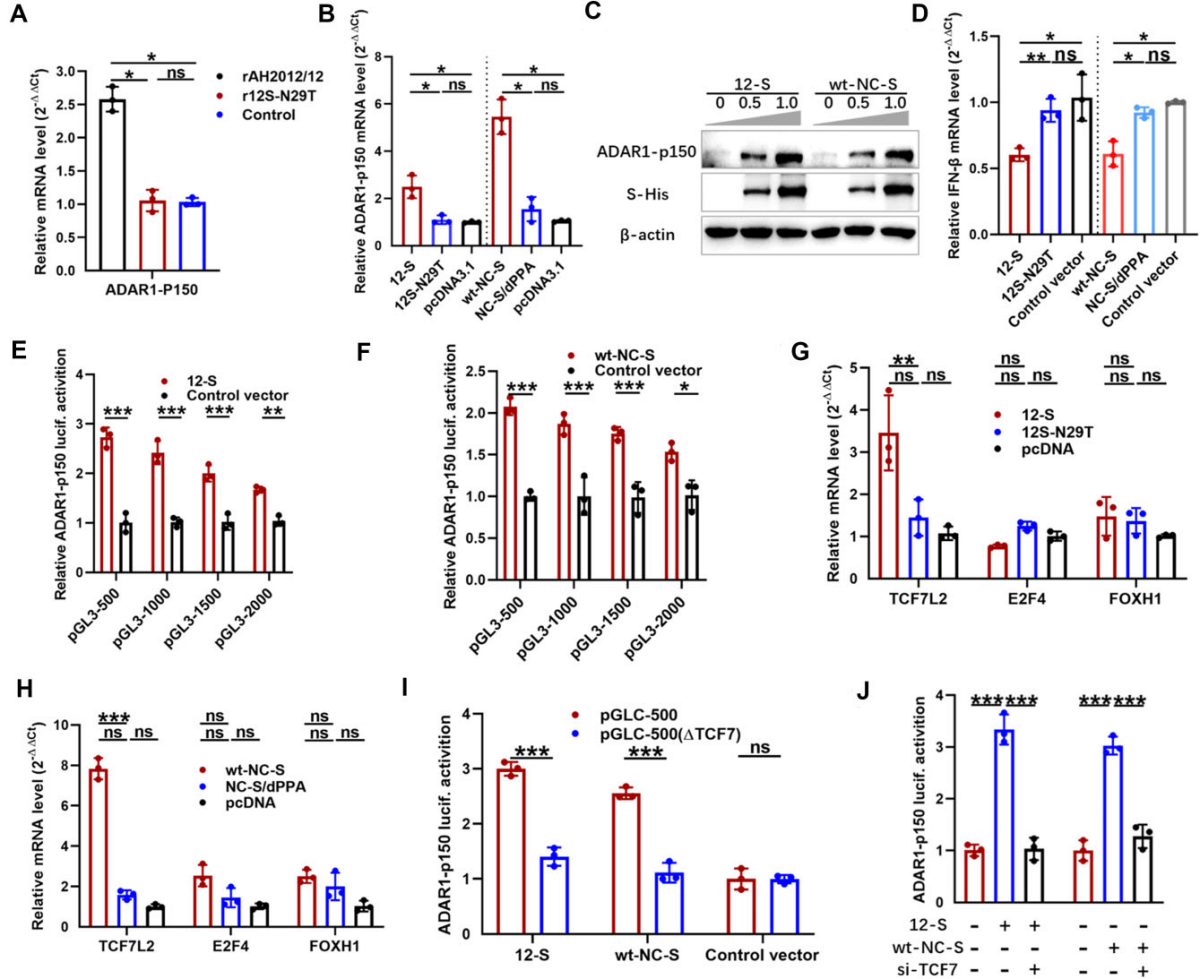
Coronavirus S transcriptionally promotes ADAR1-p150 expression via TCF7L2. The mRNA levels of ADAR1-p150 were detected in Vero cells infected with rAH2012/12 or mutant strain r12S-N29T (**A**) and in cells transfected with the S and its mutants (**B**). The protein levels of ADAR1-p150 were detected in Vero cells transfected with different doses of 12-S or wt-NC-S plasmids (**C**). Figures are representative of two independent experiments. The mRNA levels of IFN-β were detected in Marc-145 cells transfected with the S and its mutants (**D**). The ADAR1-p150 promoter activities were assessed in Vero cells transfected with 12-S (**E**) or wt-NC-S (**F**) plasmids and a series of truncated ADAR1-p150 promoter reporter plasmids. The mRNA levels of TCF7L2, E3F4 and FOXH1 were detected in Vero cells transfected the PEDV (**G**) or SARS-CoV-2 (**H**) S and its mutants. The ADAR1-p150 promoter activities were assessed in Vero cells transfected with 12-S or wt-NC-S plasmids and ADAR1-p150 promoter pGLC-500 or the promoter pGLC-500 (ΔTCF7) with the deletion of the TCF7L2-binding motif plasmids (**I**). Vero cells were pretreated with siTCF7L2, co-transfected with 12-S or wt-NC-S and ADAR1-p150 promoter reporter plasmids, and incubated for 36 h. The ADAR1-p150 promoter activity was then assessed (**J**). The bar graphs showed the data of three independent experiments, presented as mean ± SD. Statistics: Student’s *t*-test or two-way ANOVA multiple comparisons tests (*P < 0.05, **P < 0.01, ***P < 0.001).

To investigate the mechanisms by which S proteins regulate ADAR1-p150 expression, we measured the transcription levels of ADAR1-p150 in response to the parental PEDV strain and the wt and mutant S proteins of PEDV and SARS-CoV-2. The results showed that both the parental PEDV strain and the wt S proteins of both viruses significantly increased ADAR1-p150 transcription levels, but their respective mutants had no effect (Figure 8A and B). Dose-dependent experiments further confirmed positive correlations between the expression levels of ADAR1-p150 and 12-S or wt-NC-S (Figure 8C). Given that the ADAR1-p150 gene is induced by type I interferon (IFN), the Marc-145 cell line, with a complete IFN pathway, was selected for the next experiment. The results showed that, compared with the control vector transfected cells, the parental S protein significantly reduced IFN-β transcription levels, whereas the mutant 12S-N29T and NC-S/dPPA proteins had no effect (Figure 8D). Furthermore, in Marc-145 cells, overexpression of the parental S protein significantly upregulated the expression level of ADAR1-p150 and notably inhibited the formation of SGs induced by poly I:C (Supplementary Figure S5A–C). In contrast, 12S-N29T and NC-S/dPPA failed to upregulate ADAR1-p150 expression or inhibit poly I:C-induced SG formation (Supplementary Figure S5A–C). These results indicated that type I IFN is not the predominant factor through which the S protein upregulates ADAR1-p150 expression.

To elucidate the regulatory mechanism underlying the enhanced transcriptional activation of ADAR1-p150 by the S protein, truncated constructs of the 5′-flanking region of the ADAR1-p150 promoter were cloned into reporter plasmids and transfected into Vero cells, along with 12-S or wt-NC-S expression plasmids. Luciferase reporter assays showed that the –500 bp region contains the core element critical for S mediated transregulation of ADAR1-p150 (Figure 8E and F). Furthermore, ADAR1-p150 promoter activities in Vero cells expressing either 12S-N29T or NC-S/dPPA were lower than those expressing 12-S or wt-NC-S (Supplementary Figure S6). Subsequent analysis using TRANSFAC (http://biogrid-lasagna.engr.uconn.edu/lasagna_search/a) identified three main binding sites for the transcription factors TCF7L2, E3F4 and FOXH1. Overexpression of 12-S and wt-NC-S significantly upregulated TCF7L2 levels (Figure 8G and H), indicating the involvement of TCF7L2 in S-mediated promotion of ADAR1-p150 transcription. To verify this role of TCF7L2, we transfected cells with a mutant ADAR1-p150 promoter reporter plasmid lacking the TCF7L2-binding motif and TCF7L2-targeting siRNA. As shown in Figure 8I, deletion of the TCF7L2-binding motif in the ADAR1 promoter suppressed the S-mediated enhancement of ADAR1-p150 promoter activity. Consistently, TCF7L2 knockdown also dampened the S-mediated enhancement of ADAR1-p150 transcription (Figure 8J). These findings supported the hypothesis that TCF7L2 is crucial for coronavirus S-mediated transcriptional enhancement of ADAR1-p150 expression.

## Discussion

SGs form in response to some viral infections, playing a role in antiviral defense. However, most coronavirus infections do not induce the formation of SGs. This is mainly attributable to the inhibitory effects of various viral structural and non-structural proteins, including nsp1, nsp5, nsp15 and N, as well as the accessory protein 4a, on the formation of SGs (18–20,22,51–54). This coronavirus protein-mediated inhibition of SG formation facilitates viral translation, promoting efficient virus replication (27). Our findings elucidate the regulatory role of the coronavirus S protein, which serves to enhance the RNA editing activity of ADAR1-p150 and suppress PKR activity. This, in turn, affects the levels of viral dsRNA and the induction of SGs, offering novel insights into the role of coronavirus S proteins in modulating SG formation.

SGs are dynamic, large aggregates of cytoplasmic mRNA and protein. Upon infection, viruses from diverse families provoke a stress response and SG formation in host cells; however, many have evolved mechanisms to subsequently block the assembly of SGs (55). Previous studies have found that, early in infection, the alphaviruses Semliki Forest virus and poliovirus induce SG formation that declines as the infection progresses and viral replication induces transcriptional and translational shutoffs (56,57). Furthermore, it has been observed that the SARS-CoV-2 nsp5 and N protein play a role in regulating the formation of antiviral SGs (54). In this study, infection with the classical PEDV strain JS2008 significantly induced SG formation in up to 44% of cells at 24 hpi, which decreased to 21% at the late stage of infection. This indicated that 24 hpi may correspond to the peak of JS2008 replication, with the subsequent decrease in SGs potentially linked to reduced viral replication and increased viral protein levels.

ADAR1 exhibits proviral activity through two mechanisms: RNA editing and the inhibition of PKR activation. While these pathways have been studied independently, they synergistically promote viral replication and contribute to viral pathogenesis (58). To screen for coronavirus proteins that inhibit SG formation, we employed SA as a positive stimulus. SA induces SG formation by activating PKR through the heat shock response, independently of dsRNA (59). Hence, the impact of ADAR1-p150, which directly inhibits PKR activity, was examined. Such inhibitory effects may lead to the inhibition of PKR activation in AH2012/12 infection, which induces a limited amount of dsRNA. Furthermore, the S protein and its variants regulate ADAR1-p150 expression differently, thereby affecting their inhibition of SG formation.

Viral infections often generate long, uninterrupted dsRNAs, a characteristic that is relatively rare in cellular processes. These dsRNA duplexes can be subject to hyper-editing by ADAR1, followed by specific cleavage by a ribonuclease present in various cells (60,61). In this study, we observed that the wt S protein of the PEDV mutant strain and early SARS-CoV-2 strains increased ADAR1-p150 expression, significantly reducing dsRNA levels in the cytoplasm. Furthermore, the adenosine-to-inosine editing activity of ADAR1-p150 was found to play a key role in regulating dsRNA levels. These findings support a proposed mechanism in which the elimination of exogenous dsRNA from cells involves ADAR1-mediated hyper-editing of dsRNA, followed by targeted cleavage.

Recent studies suggest that dsRNA can arise from various disrupted cellular processes, eliciting innate immune responses akin to those seen in virus-infected cells (62). The J2 antibody can be used to detect both viral and endogenous dsRNAs (63). Hence, our study considers the possibility of detecting endogenous dsRNA. However, given the essential role of dsRNA in PEDV replication and the typically low levels of dsRNA in healthy cells, we focused on identifying dsRNA following PEDV infection, predominantly of viral origin. Viral dsRNA commonly initiates multiple cellular antiviral defense mechanisms, thus decreasing viral dsRNA levels in the cytoplasm attenuates the activation of downstream antiviral pathways, enabling the virus to effectively evade host immune responses.

Coronavirus infection typically results in the formation of syncytia through cell–cell fusion, a process facilitated by the viral S protein interacting with receptors on neighboring cells. Syncytia formation may contribute to pathology by facilitating viral dissemination, cytopathicity, immune evasion and inflammatory responses (64). In this study, we observed that AH2012/12 infection induced syncytia but JS2008 did not, consistent with previous studies (65). The key region for coronavirus-induced syncytial formation was found to be located in S2. The recombinant virus r12S-T29N, carrying a mutation in the N-terminus of S, retained the syncytia-inducing capability of the parent strain AH2012/12. However, infection with r12S-T29N also led to significant increases in both dsRNA and SGs compared with infection with AH2012/12, indicating that syncytia formation may not play an important role in regulating dsRNA and SGs during coronavirus infection.

The two isoforms of ADAR1, p150 and p110, are both capable of editing dsRNA, with over half of the A-to-I editing sites selectively edited by ADAR1-p150 (37). The coronavirus S protein specifically induced high-level expression of ADAR1-p150, reducing the dsRNA produced by viral infection to a significant extent. Notably, both wt S overexpression and mutant virus infection induced ADAR1-p150 expression, with an increase in ADAR1-p110 protein detected via western blotting. However, this increase in ADAR1-p110 was more significant in virus-infected cells (Figure 6D). Previous studies have demonstrated that leaky ribosome scanning downstream of the ADAR1-p150 start codon results in the co-expression of ADAR1-p110 alongside ADAR1-p150 from the canonical ADAR1-p150-encoding mRNA. The strong Kozak consensus sequence surrounding the ADAR1-p110 start codon suggests that the mRNA encoding ADAR1-p150 is specifically designed to facilitate the co-expression of ADAR1-p110 (37). This may explain why ADAR1-p150 induction also increased ADAR1-p110 expression. Additionally, expression of the *ADAR1* gene is controlled by four alternative promoters: promoter exons 1B, 1C and 2 drive the expression of ADAR1-p110, while promoter exon 1A controls the expression of ADAR1-p150. Compared with the overexpression of the S protein, there were viral nucleic acids and other highly expressed viral proteins present after viral infection. These additional factors may activate the transcription and translation of ADAR1-p110, leading to a significant upregulation of ADAR1-p110 after viral infection. Furthermore, ADAR1-p150 gene expression is stimulated by type I IFN (30). However, in this study, S protein played an important regulatory role in ADAR1-p150 expression in IFN-deficient Vero cells, indicating that there is an additional pathway for its regulation of ADAR1-p150. Indeed, the transcription factor TCF7L2 was identified as a player in the coronavirus S protein-mediated transcriptional enhancement of ADAR1-p150 expression.

The continuous genetic variability of coronaviruses leads to the emergence of new variants, impacting immunomodulation and pathogenicity. The S protein shows significant variation due to host immune pressure (66). Compared with the classical strain, the circulating mutant PEDV strain shows higher pathogenicity (10), potentially attributable to the T29N mutation in S protein, which creates a glycosylation site and induces ADAR1-p150 expression. This results in reduced dsRNA accumulation, weakening host induction of IFN and IFN-stimulated genes. The recent 27PPA29 deletion in the N-terminus of the S proteins of SARS-CoV-2 variants, such as Omicron, eliminates ADAR1-p150 induction, possibly reducing virulence (67). The N-termini of PDCoV and TGEV S proteins have remained relatively conserved, possibly because of the low epidemic rates of PDCoV and TGEV (68) and consequent avoidance of further immune selection pressure. We speculated that the frequency of epidemics and host immune pressure are prerequisites for N-terminal variation of the S protein. Importantly, given the ongoing mutation of coronavirus genomes, particularly the high mutation rate of S, the possibility that similar mutant strains of other coronaviruses will emerge is an important area of surveillance and vigilance.

In conclusion, this study has revealed the critical role of the coronavirus S protein in the mechanism and regulation of SG formation in host cells. Following coronavirus infection, the S protein was shown to upregulate the expression of ADAR1-p150, which mediates A-to-I editing of dsRNA through its catalytic activity, leading to ribonuclease-mediated cleavage of the edited dsRNA and reduced intracellular levels of viral dsRNA. Additionally, ADAR1-p150 suppressed PKR activation, thereby inhibiting the PKR-eIF2α pathway induced by excessive virus-generated dsRNA, as well as the induction of translation arrest and SG formation. However, the continuous mutation of the coronavirus S protein has altered its regulatory effect on ADAR1-p150. Specifically, the highly variable region adjacent to the signal peptide at the N-terminus of the S protein was identified as the key region involved in regulating the expression of ADAR1-p150.

## Supplementary Material

gkae921_Supplemental_File

## Data Availability

The data underlying this article are available in the article and in its Supplementary material.
