# Supplementary material for: Coronavirus S protein alters dsRNA accumulation and stress granule formation through regulation of ADAR1-p150 expression
Source: Nucleic Acids Res. 2024 Oct 24;52(21):13174–91. doi: 10.1093/nar/gkae921 (PMC11602127; doi:10.1093/nar/gkae921)
Supplement: gkae921_Supplemental_File [file gkae921_supplemental_file.pdf]

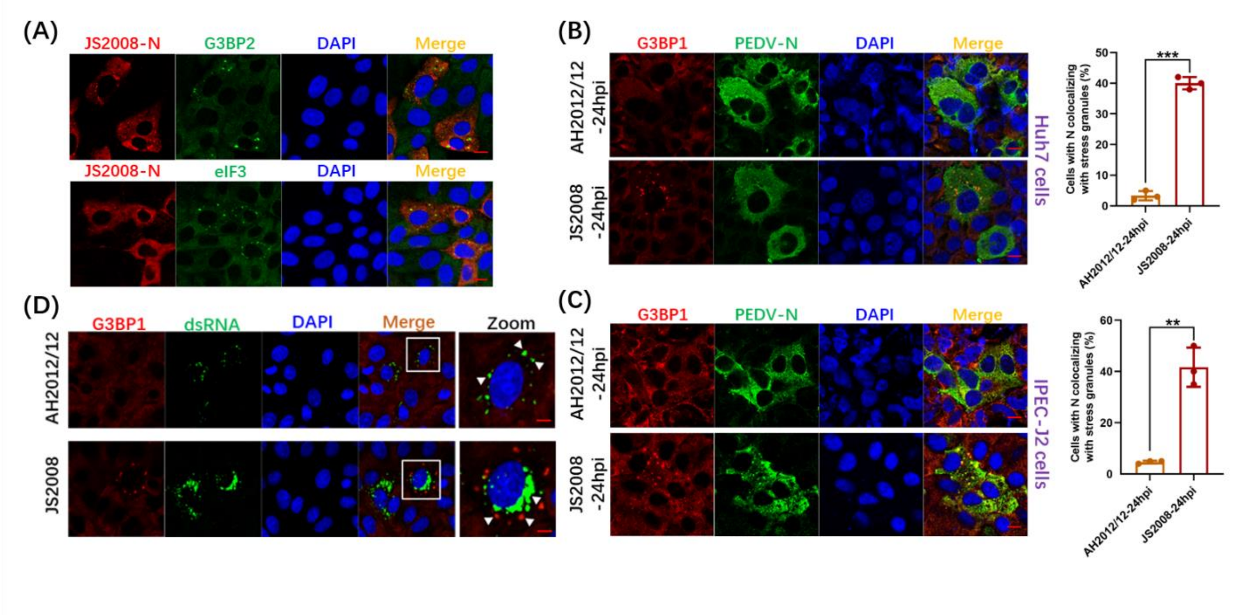

Figure S1. Compared with the classical strain, the mutant PEDV infection produces less dsRNA and inhibits SG formation in an eIF2α-dependent manner. Vero cells were infected with JS2008. At 12 hpi, immunostaining was used to visualize the SGs using anti-PEDV-N (red) with anti-G3BP2 (green), or anti-eIF3 (green) (A). IPEC-J2 (B) and Huh7 (C) cells were incubated with AH2012/12 or JS2008 at 1 MOI. At 24 hpi, cells were immunostained with anti-G3BP1 (red) and anti-PEDV-N (green) antibodies. The percentages of SGs-positive cells relative to infected cells of three independent experiments were displayed in bar graphs. Vero cells were infected with AH2012/12 or JS2008, and the cells were immunostained with dsRNA (green) and G3BP1 (red) (D). For (A)-(D), the images are representative of three independent experiments. Data are shown as the mean ± SD. Statistics: Student t test (**, $p < 0.01$; ***, $p < 0.001$). Scale bars: 10 μm.

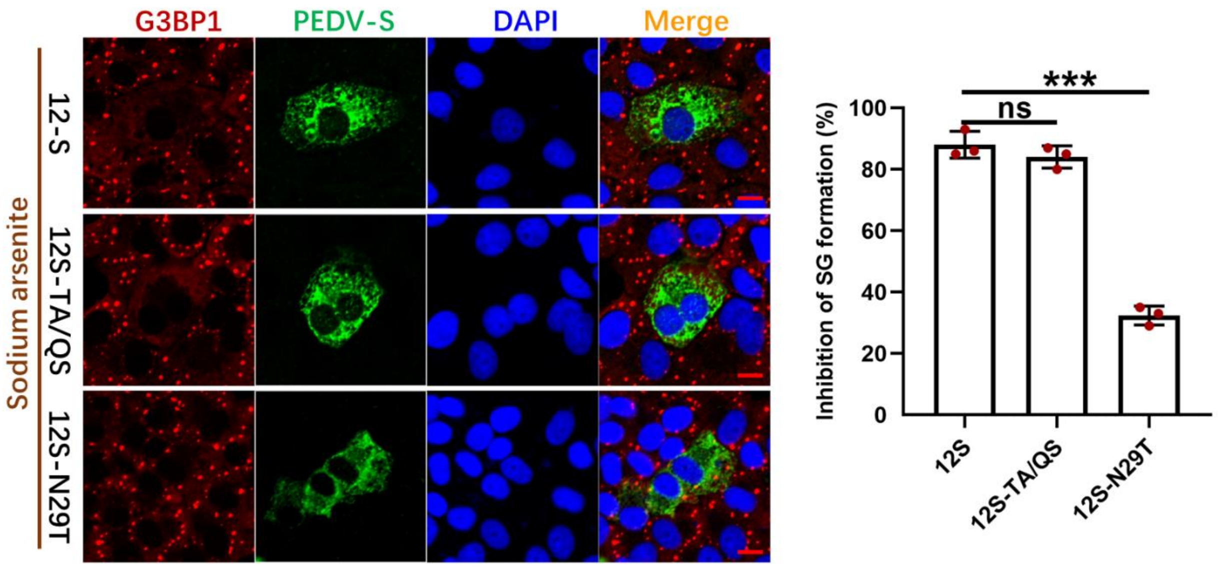

Figure S2. The 29th amino acid of the mutant PEDV S protein is critical for inhibiting SG formation induced by SA. Vero cells were transfected with 12-S, 12S-TA/QS and 12S-N29T expressed plasmids. At 24 h post-transfection, cells were treated with SA, and then were immunostained with anti-G3BP1 (red) and anti-PEDV-S (green) antibodies. The images are representative of three independent experiments. The percentages of inhibition of SG formation relative to S expressed cells of three independent experiments were displayed in bar graph. Data are shown as the mean ± SD. Statistics: Student t test (***, $p < 0.001$). Scale bars: 10 μm.

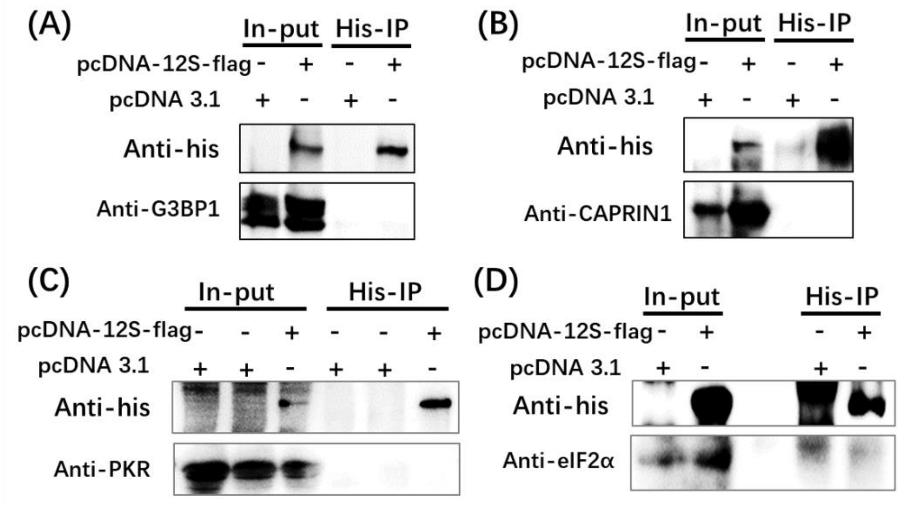

Figure S3. The immunoprecipitation assay of AH2012/12 S protein with G3BP1, CAPRIN1, PKR, and eIF2α. Vero cells were transfected with 12-S recombinant plasmid. After 24 hours, the cells were lysed in protein extraction reagent for 30 min on ice, followed by centrifugation at 10,000×g for 10 min at 4°C to remove cell debris. Then the cell lysates were then incubated with mouse anti-flag antibodies for 12 hours at 4°C, then A/G-agarose beads (Beyotime) were added. After 4 h incubation, the beads were collected by centrifugation at 2,500 g for 5 min and washed five times with cold PBS. The beads were boiled in 2×SDS loading buffer to elute bound protein of G3BP1 (A), CAPRIN1 (B), PKR (C), and eIF2α (D) with corresponding antibodies, respectively. And the figures are representative of two independent experiments.

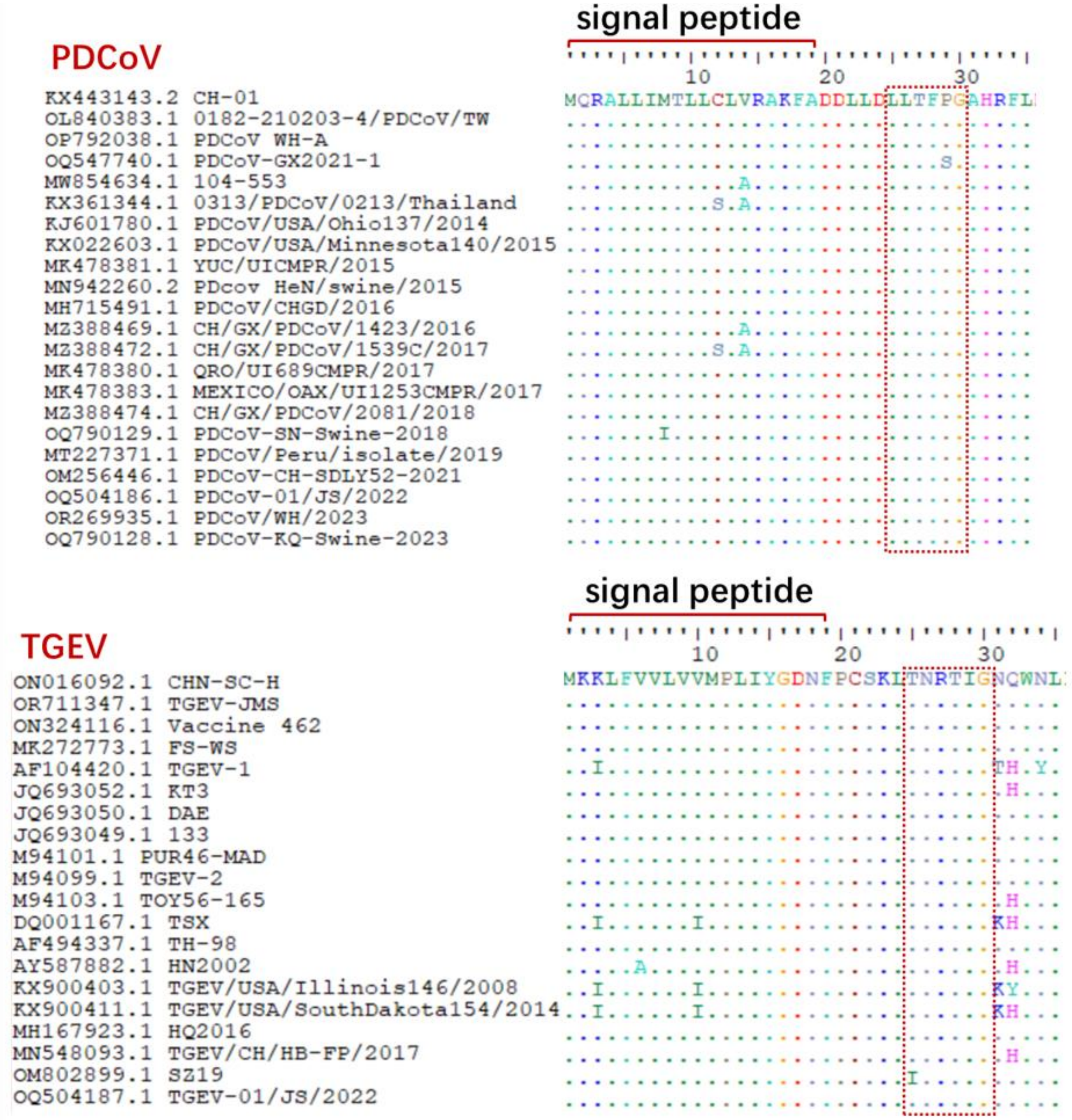

Figure S4. Amino acid sequence alignment of the N-terminals of PDCoV and TGEV strains isolated in different years.

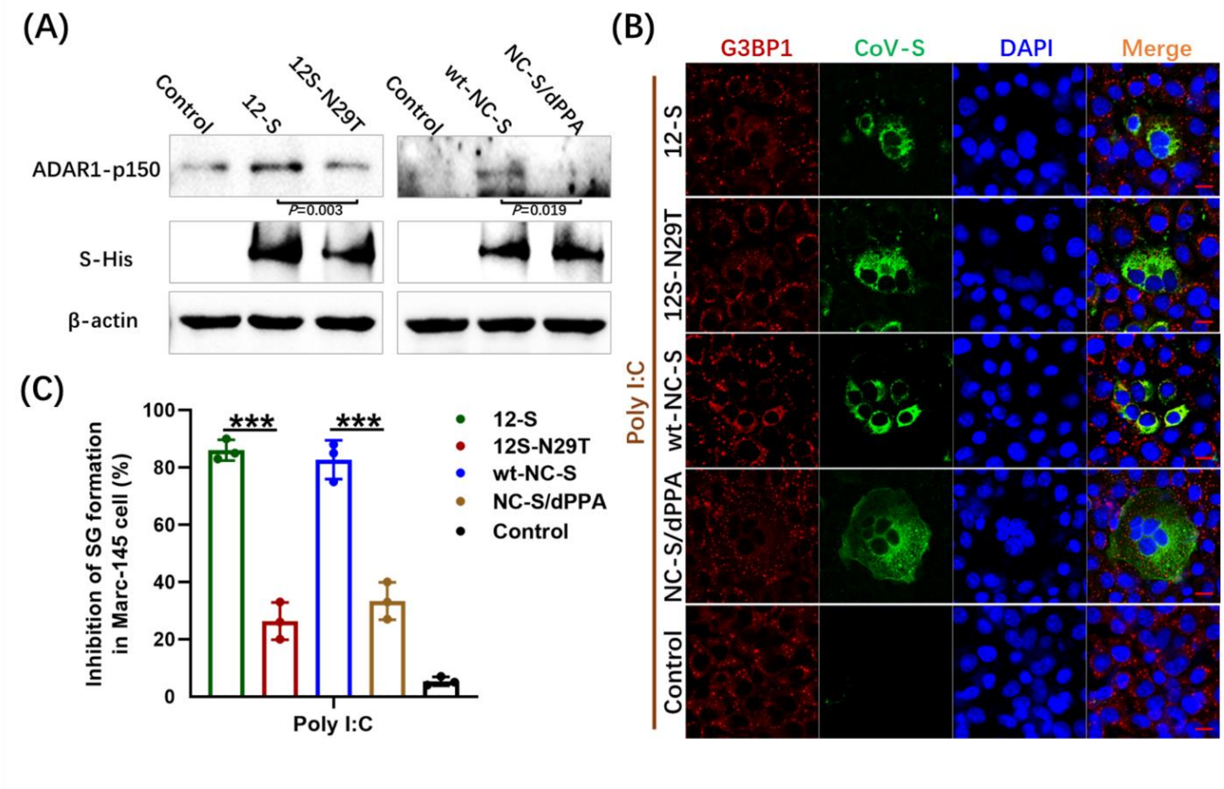

Figure S5. The effects of S and the mutants on the expression levels of ADAR1-p150 and the inhibition of SGs induced by Poly I:C in Marc-145 cells. Marc-145 cells were transfected with 12-S or 12S-N29T, and wt-NC-S or NC-S/dPPA. At 24 h post-transfection, the expression levels of ADAR1-p150, S, and β-actin were detected (A). Figures are representative of two independent experiments. The relative expression levels of target proteins ADAR1-p150 were determined by measuring grayscale of β-actin in cells using Image J. Marc-145 cells were transfected with S or the mutant plasmids. At 18 h post-transfection, the cells were transfected with poly I:C for six hours, then were fixed and incubated with anti-His (green) and anti-G3BP1 (red) antibodies (B). The images are representative of three independent experiments. The percentages of inhibition of SGs formation relative to S expressed cells of three independent experiments were displayed in bar graph (C). Data are shown as the mean ± SD. Statistics: Student t test (***, $p < 0.001$). Scale bars: 10 μm.

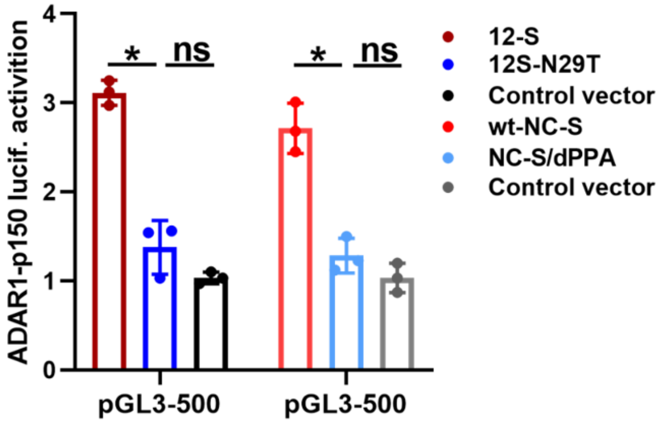

Figure S6. Detection of ADAR1-p150 promoter activities in Vero cells transfected with PEDV or SARS-CoV-2 S and the mutant plasmids and the ADAR1-p150 promoter reporter plasmids. The bar graph showed the data of three independent experiments, presented as mean ± SD. Statistics: two-way ANOVA multiple comparisons tests (*, $p < 0.05$).

Table S1. The sequences of primers used in this study.

Name	Sequence (5'-3')	Note
Primers of recombinant pcDNA3.1 of PEDV proteins.		
NSP1-F	*CTTGGTACCGAGCTCGGATCC*GCCACCA TGGCTAGCAACCATGTTACATTGGCTG	
NSP1-R	*GCACAGTGGCGGCCGCTCGAG*TTA CTTATCGTCGTCATCCTTGTAATC ACCACCACGACGACCAAAAGTGA	
NSP4-F	*CTTGGTACCGAGCTCGGATCC*GCCACCA TGGCAGGTTTTCCTAGTTTTTCAAAGG	
NSP4-R	*GCACAGTGGCGGCCGCTCGAG*TTA CTTATCGTCGTCATCCTTGTAATC CTGTAGAGTTGAATTGTAACTCA	
NSP5-F	*CTTGGTACCGAGCTCGGATCC*GCCACCA TG GCTGGCTTGCGTAAGATGGCACAAC	
NSP5-R	*GCACAGTGGCGGCCGCTCGAG*TTA CTTATCGTCGTCATCCTTGTAATC CTGAAGATTAACGCCATACATTT	The italic represents recombination sequence. The underline represents flag tag sequence.
NSP6-F	*CTTGGTACCGAGCTCGGATCC*GCCACCA TG AGTGGTTATGTTTCACGCGCCTGCA	
NSP6-R	*GCACAGTGGCGGCCGCTCGAG*TTA CTTATCGTCGTCATCCTTGTAATC CTGAACGGAAGAAATCTTAATAT	
NSP7-F	*CTTGGTACCGAGCTCGGATCC*GCCACCA TG TCTAAATTGACTGATATTAAGTGTA	
NSP7-R	*GCACAGTGGCGGCCGCTCGAG*TTA CTTATCGTCGTCATCCTTGTAATC ACTCTGCAACATACTATTGTCAT	
NSP8-F	*CTTGGTACCGAGCTCGGATCC*GCCACCA TG GTTGCATCTACTTATGTTGGTTTGC	
NSP8-R	*GCACAGTGGCGGCCGCTCGAG*TTA CTTATCGTCGTCATCCTTGTAATCCTGG AGCTTGACAATACGCTCAC	

NSP9-F	*CTTGGTACCGAGCTCGGATCC*GCCACCATG AATAATGAAATTATTCCTGGTAAGC
NSP9-R	*GCACAGTGGCGGCCGCTCGAG*TTA CTTATCGTCGTCATCCTTGTAATCCTGC AAGCGTACAGTGGCACCTA
NSP10-F	*CTTGGTACCGAGCTCGGATCC*GCCACCATG GCTGGTAAACAAACAGAACAGGCTA
NSP10-R	*GCACAGTGGCGGCCGCTCGAG*TTA CTTATCGTCGTCATCCTTGTAATCTTGC ATAATGGATCTGTCACAAG
NSP12-F	*CTTGGTACCGAGCTCGGATCC*GCCACCATG GCTTATTTAAACGAGTACGGGG
NSP12-R	*GCACAGTGGCGGCCGCTCGAG*TTA CTTATCGTCGTCATCCTTGTAATCATT GCAAAACTGCAGATTTCTCA
NSP13-F	*CTTGGTACCGAGCTCGGATCC*GCCACCATG TCTGCAGGGCTTTGTGTTGTTTGTG
NSP13-R	*GCACAGTGGCGGCCGCTCGAG*TTA CTTATCGTCGTCATCCTTGTAATCCTG CAAATCAGACAATTTAAGCT
NSP14-F	*CTTGGTACCGAGCTCGGATCC*GCCACCATG GCTAATGAGGGTTGTGGTCTTTTTA
NSP14-R	*GCACAGTGGCGGCCGCTCGAG*TTA CTTATCGTCGTCATCCTTGTAATCTTGC AAATTGTTACTAAATGTCT
NSP15-F	*CTTGGTACCGAGCTCGGATCC*GCCACCATG GGCCTTGAGAACATTGCTTTTAATG
NSP15-R	*GCACAGTGGCGGCCGCTCGAG*TTA CTTATCGTCGTCATCCTTGTAATC CTGAAGTTGCGGATAAAATGTCT
NSP16-F	*CTTGGTACCGAGCTCGGATCC*GCCACCATG GCCAGTGAATGGAAGTGTGGTTATT

NSP16-R	*GCACAGTGGCGGCCGCTCGAG*TTA CTTATCGTCGTCATCCTTGTAATCTTTG TTTACGTTGACCAAATGAT
AH12-ORF3-F	*CTTGGTACCGAGCTCGGATCC*GCCACCA TGGTTCTTGGACTTTTTCAATACACGA TT
AH12-ORF3-R	*GCACAGTGGCGGCCGCTCGAG*TTA CTTATCGTCGTCATCCTTGTAATCCACT GCACGTGGACCTTTTCAAAAGCTTCG
JS08-ORF3-F	*CTTGGTACCGAGCTCGGATCC*GCCACCA TGGTTCTTGGACTTTTTCAATACACGA TT
JS08-ORF3-R	*GCACAGTGGCGGCCGCTCGAG*TTA CTTATCGTCGTCATCCTTGTAATCAAC AAAGCCTGCCAATAAGTGTGCA
E-F	*CTTGGTACCGAGCTCGGATCC*GCCACCA TGGTACAATTAGTGAATGATAATGGT CTAG
E-R	*GCACAGTGGCGGCCGCTCGAG*TTA CTTATCGTCGTCATCCTTGTAATCACTC GTCTAGTTGAATTGAATCAAATGCAG
M-F	*CTTGGTACCGAGCTCGGATCC*GCCACCA TGGCTAACGGTTCTATTCCCGTTGATG AG
M-R	*GCACAGTGGCGGCCGCTCGAG*TTA CTTATCGTCGTCATCCTTGTAATCATTT CGTTTATACGTCAATAACAGTACTGG
N-F	*CTTGGTACCGAGCTCGGATCC*GCCACCA TGGCTTCTGTCAGTTTTCAGGATCGTG GC
N-R	*GCACAGTGGCGGCCGCTCGAG*TTA CTTATCGTCGTCATCCTTGTAATCAAA GTTTCTGTTTAGACTAAATGAAGCACT
Primers of PEDV S mutants.	

08-S1-F	GCACAGCTGGCGTGCTGAGC<br>CGGTTCTTTTCAAAATTTAATGTTCAG<br>GCA
08-S1-R	GGGGCCTCTGGGATGTATCC<br>ATACACCAACACAGGCTCTGTACAATT<br>GGA
12S1-F	GCACAGCTGGCGTGCTGAGC<br>CGGTTCTTTTCAAAATTTAATGTTCAG<br>GCG
12S1-R	GGGGCCTCTGGGATGTATCC<br>ATACACCAACACAGGCTCTGTACAATT<br>AGA
12S1-qN231-F	GCACAGCTGGCGTGCTGAGC<br>GCTAATTGCATTGGTTATGCTGCCAAT<br>GTA
12S1-qC504-R	GGGGCCTCTGGGATGTATCC<br>AGTTGGCTGTTCATGACTCAGAAGGTT<br>TGT
CTDzl-R	TAATATTAACAAAAGAATGATCATT<br>GAAGCTAGGGAGGGTCACGAAGCTGG<br>TTGG
CTDzl-F	AGCTTTTGAAAAGGTCCACGTGCAG<br>GGATACATCCCAGAGGCCCCGCGGGA<br>CGGC
08CTD-F	AATGATCATTCTTTTGTTAATATTACTG<br>TC
08CTD-R	CTGCACGTGGACCTTTTCAAAAGCTTC<br>GTA
12/08-I-F	CAGTCTACTATT<br>AACTTTAGACGGTTCTTCAGCAAATTC<br>AAC

12/08-I-R	TCTAAAGTTAATAGTAGACTG GCATCTAGTGACATCCTGAGGGAGGCT CAG
12/08-II-F	AGTATGAACTCTTCTAGC TGGTACTGCGCCGGCCAACACCCCACA GCC
12/08-II-R	ACCAGCTAGAAGAGTTCATACT AGGCAGGTAGCCCCCCAGCACCACCA CAGC
12/08-III-F	GGCACAGGCATTGAA ACAGCCAGCGGCGTGCACGGTATCTTC CTG
12/08-III-R	TGGCTGTTTCAATGCCTGTGCC GCAGTACCAGGTGGAGTTCACGCCCTG GTT
12/08-IV-F	TACATCGATTCTGGTCAG GGCTTCGAGATCGGCATCAGCCAGGA GCCC
12/08-IV-R	AGCCCTGACCAGAATCGATGTA GCTCAGGAAGATACCGTGCACGCCGCT GGC
12/08-V-F	GATAAT AAGACGCTGGGCCCTGCCGCTAACAA CGAC
12/08-V-R	CAGGGCCCAGCGTCTTATTATC AGGAAACTGGCAGATCCGCAGTCTGG CCGT
12/08-VI-F	TTGCAGGATGGAAAAAATATT GTGGTGGGAATCACCTGGGACAACGA CAGA

12/08-VI-R	AATATTTTTTCCATCCTGCAA GGCGGGGATGGCTTTGTTGAACAGAC AATT
12/08-VII-F	AAAAGAAGT TGTGCCATGCAGTACGTGTACGAGCCT ACC
12/08-VII-R	ACTGCATGGCACAACTTCTTTT GTTGTAGCATTTGGTGGCCACCCGGCT CCA
12/08-QS-F	CagtctAATACGAACTTTAGACGGTTCTT CAGCAAATTCAAC
12/08-QS-R	TCTAAAGTTCGTATTagactgGCATCTAG TGACATCCTGAGGGAGGCTCAG
12/08-N29T-F	ACCGCCactaCGAACTTTAGACGGTTCTT CAGCAAATTCAAC
12/08-N29T-R	TCTAAAGTTCGtagtGGCGGTGCATCTAG TGACATCCTGAGGGAGGCTCAG
Primers of ADAR1-p150 mutants.	
ADAR1-qZa-F	GCTCAGGCTTGGAACCAGCACAGCGG AGTGGT
ADAR1-qZa-R	TGCTGGTTCCAAGCCTGAGC CATAAGCTTGCCGCCGGACTTGTACAG CTCGT
ADAR1-qZaZb-F	AAGAACCCCATCAGCGGGCTGTTAGA ATATGCCCA
ADAR1-qZaZb-R	AGCCCGCTGATGGGGTTCTT CATAAGCTTGCCGCCGGACTTGTACAG CTCGT

ADAR1-H910Q/E912A-F	GCCAAGCAGCAATAATCTCCCGGAGAGGCTTCATCA
ADAR1-H910Q/E912A-R	GGGAGATTATTGCTGCTTGGCAGTCATTGACAGTTTCTCCTT
Primers of SARS-CoV-2 S mutants.	
NC-S-qPPA-F	TTACTACTAGAACCCAGCTCTATACCAACTCTTTCACCAGGGGCGTATATTACC
NC-S-qPPA-R	GAGCTGGGTTCTAGTAGTAAGGTTGACGCA
Primers of recombinant virus construction.	
sgRNAPEDV08IF	TTCTAATACGACTCACTATAGGCTTCTTTCCCTATATTAAGTTTTAGAGCTAGA
sgRNAPEDV08IR	TTCTAATACGACTCACTATAGGGCCCAATGTTTTAATGCTGTTTTAGAGCTAGA
PEDV08IF	AAGAAGGCTTCTTTCCCTATAT
PEDVD0upR	AATAGTAGACTGGCACCTGGTGACATCTTGTGGT
PEDVD0DownF	CACCAGGTGCCAGTCTACTATTAATTTTAGGCGGTTCTTTTC
PEDV08IR	CAGTGGGGCCCAATGTTTTAAT
Scaffold oligo	AAAAGCACCGACTCGGTGCCACTTTTTCAAGTTGATAACGGACTAGCCTTATTTTAACTTGCTATTTCTAGCTCTAAAA
Primers of ADAR1-p150 promoter reporter construction.	
p150+300-R	CCCGACCCGCCGGCGGCACGACCCT
p150-500-F	CCCGACCCGCCGGCGGCACGACCCT
p150-1000-F	TGCACTCCAGCCTGGGCGACAGAGC
p150-1500-F	ATGTGCAAAGGCCCAGAAGGAAGAC
p150-2000-F	TGTCTTTCGATTGTTTTGCCCTTGT

Primers of qRT-PCR.	
rtIFN-β-F	TGCATCCTCCAAATCGCTCT
rtIFN-β-R	ATTGAGGAGTCCCAGGCAAC
mADAR1-p150-F	ACTTTCCGGAGGGGAAGGCT
mADAR1-p150-R	GCGTCGGCACAGGAAACTCT
mTCF7L2-F	GGGAAACCAACGAACACAGC
mTCF7L2-R	GCGTATTTGCATCTGCAGGG
mE2F4-F	AAGCCTGCCTTAGCTCAACC
mE2F4-R	GGCTACCACTCACTGCAATC
mFOXH1-F	TTGCTGACCGAATGGGGGAA
mFOXH1-R	CCGCCCAGAAGTTACCCTTG
